# Polycaprolactone Adsorption and Nucleation onto Graphite Nanoplates for Highly Flexible, Thermally Conductive, and Thermomechanically Stiff Nanopapers

**DOI:** 10.1021/acsami.1c16201

**Published:** 2021-12-01

**Authors:** Kun Li, Daniele Battegazzore, Ricardo A. Pérez-Camargo, Guoming Liu, Orietta Monticelli, Alejandro J. Müller, Alberto Fina

**Affiliations:** † Dipartimento di Chimica e Chimica Industriale, Università di Genova, Via Dodecaneso 31, 16146 Genova, Italy; ‡ Dipartimento di Scienza Applicata e Tecnologia, Politecnico di Torino-Alessandria Campus, viale Teresa Michel, 5, 15121 Alessandria, Italy; § Beijing National Laboratory for Molecular Sciences, Institute of Chemistry, Chinese Academy of Sciences, 100190 Beijing, China; ∥ University of Chinese Academy of Sciences, 100049 Beijing, China; ⊥ POLYMAT and Department of Polymers and Advanced Materials: Physics, Chemistry and Technology, Faculty of Chemistry, University of the Basque Country UPV/EHU, Paseo Manuel de Lardizabal 3, 20018 Donostia-San Sebastián, Spain; # Basque Foundation for Science, IKERBASQUE, 48009 Bilbao, Spain

**Keywords:** polycaprolactone/graphite nanoplates nanopapers, oriented polycaprolactone, pre-freezing effect, thermomechanical properties, thermal conductivity, graphene-related materials

## Abstract

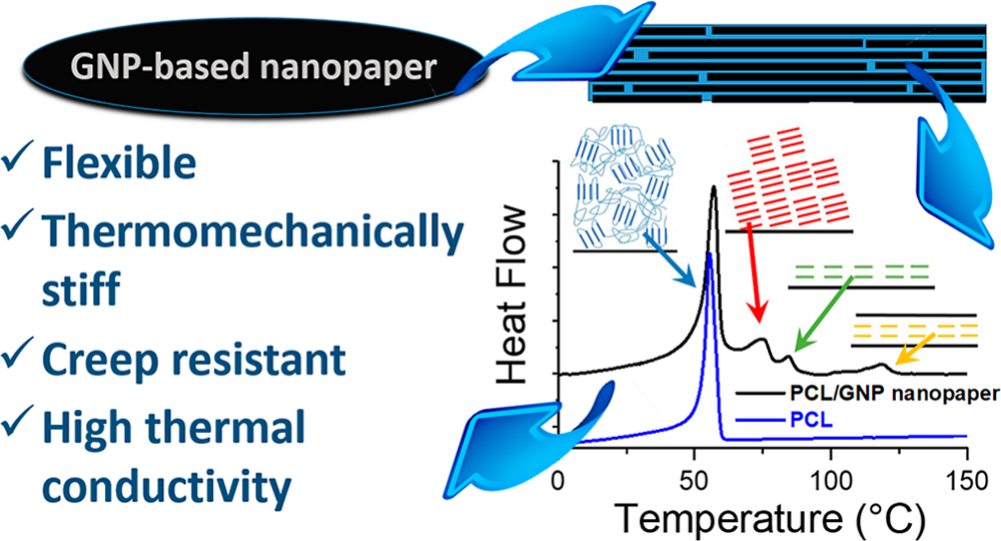

Free-standing nanopapers based on graphene and its related materials have been widely studied and proposed for flexible heat spreader applications. Given that these materials are typically brittle, this work reports the exploitation of polycaprolactone (PCL) as a polymer binder to enhance resistance and flexibility of nanopapers based on graphite nanoplates (GNP), while maintaining a high thermal conductivity. Properties of nanopapers appear to correlate with the excellent PCL adhesion and strong nucleation of the surface of GNP flakes. Furthermore, different crystalline populations were observed for PCL within the nanopaper and were investigated in detail via differential scanning calorimetry advanced techniques and X-ray diffraction. These demonstrated the coexistence of conventional unoriented PCL crystals, oriented PCL crystals obtained as a consequence of the strong nucleation effect, and highly stable PCL fractions explained by the formation of crystalline pre-freezing layers, the latter having melting temperatures well above the equilibrium melting temperature for pristine PCL. This peculiar crystallization behavior of PCL, reported in this paper for the first time for a tridimensional structure, has a direct impact on material properties. Indeed, the presence of high thermal stability crystals, strongly bound to GNP flakes, coexisting with the highly flexible amorphous fraction, delivers an ideal solution for the strengthening and toughening of GNP nanopapers. Thermomechanical properties of PCL/GNP nanopapers, investigated both on a heating ramp and by creep tests at high temperatures, demonstrated superior stiffness well above the conventional melting temperature of PCL. At the same time, a thermal conductivity > 150 W/m·K was obtained for PCL/GNP nanopapers, representing a viable alternative to traditional metals in terms of heat dissipation, while affording flexibility and light weight, unmatched by conventional thermally conductive metals or ceramics. Besides the obtained performance, the formation of polymer crystals that are stable above the equilibrium melting temperature constitutes a novel approach in the self-assembly of highly ordered nanostructures based on graphene and related materials.

## Introduction

Nanopapers are thin sheets or films composed of self-assembled individual nanoparticles, generally obtained by filtration of a suspension in a solvent. They have gained increasing interest for their unique features, such as mechanical properties, gas barrier, and flame retardancy.^1−6^ Indeed, the above properties are mainly related to the highly concentrated nanoparticles, which are tightly packed in the thin films, because of their strong self-interactions^7,8^ or mediated through a binding polymer, in the so-called brick and mortar structures.^9−14^ Among the different lamellar nanoparticles which can be exploited in the preparation of nanopapers, graphene-related nanomaterials (GRMs), such as graphite nanoplates (GNP) and multilayer graphene, represent ideal systems for producing high-performance nanopapers, being characterized by ultrahigh strength, excellent electrical, and thermal conductivity.^15−19^

Concerning the preparation of GRM nanopapers, two issues have to be considered, which are the dispersion or exfoliation to individual graphene nanosheets in a medium and the strong bonding among nanosheets in the resulting nanopapers. To overcome the dispersion problem, covalent functionalization of graphene has been usually exploited.^17,20−24^ Huang et al.^17^ prepared graphene nanopapers by flow-directed assembly starting from benzenesulfonic acid functionalized graphene nanosheets, which facilitated the dispersion of graphene nanosheets in water and allowed the preparation of nanopapers. Similarly, Korkut et al.^25^ produced a graphene network by tape-casting surfactant-stabilized aqueous suspensions of functionalized graphene sheets, followed by the removal of the polymer matrix and the surfactant. An alternative strategy to promote graphene dispersion consists in the application of graphene oxide (GO),^26,27^ which, containing hydroxyl and epoxy groups on the basal planes and carboxyl groups on the edges, results to be easily dispersible in water.^26,28−30^ By applying this approach, Chen et al.^16^ prepared graphene papers starting from GO dispersions, which were subsequently reduced with hydrazine. Indeed, the above reduction step is essential to restore the conductivity properties of the material but represents a limitation of the method as the complete reduction of GO can hardly be achieved and various defects remain within the sp^2^ carbon structure of reduced GO (rGO).^31−35^

The major drawbacks of the GRM nanopapers, particularly of those made of GNP, are their limited toughness and deformability, which is mainly related to the scarce bonding between nanosheets. Indeed, as reported in the literature, the elongation at break is typically less than 1.0%, thus limiting the practical applications of nanopapers.^16,17,25,36^ As such, the incorporation of a limited amount of polymers into the GNP nanopapers, in a brick and mortar organization, may enhance their toughness and deformability. However, the presence of a soft and non-conductive polymer between GNP is clearly expected to decrease the thermal and electrical conductivity of the nanostructure.

Building on the current state of the art, the design of organic/inorganic GNP-based nanopapers was addressed in this work. In particular, to preserve thermal conductivity of the nanopapers, highly crystalline polymers should be used^37−44^ while, to enhance the final ductility of the nanopaper, sufficient polymer mobility should be granted. With this scope in mind, polycaprolactone (PCL) was selected and exploited for the first time in GNP nanopapers, based on its high crystallinity and capability of strong nucleation on carbon nanostructures^45^ coupled with a low glass transition temperature. Furthermore, PCL is a biodegradable and biocompatible polymer, in principle allowing for application of the GNP/polymer system also in the biomedical field.^46−48^ GNP/PCL nanopapers were prepared with a polymer content ranging from 5 to 20 wt % by applying a solution blending approach, followed by filtration, drying, and pressing treatments. The nanopapers demonstrated a set of performance suitable for application in flexible heat exchangers, including flexible electronics,^49^ low temperature heat recovery,^50,51^ and wearable and implantable devices.^52,53^

## Experimental Section

### Materials

PCL is a commercial product purchased from Perstorp UK (Capa6500, *M*_n_ = 50,000 g/mol). GNP used in this work is supplied by AVANZARE (Navarrete, La Rioja, Spain) prepared via rapid thermal expansion of overoxidized-intercalated graphite, as previously reported^54^ and used as supplied without any further treatments. Detailed characterization of this GNP grade, including X-ray photoelectron spectroscopy, Raman spectroscopy, specific surface area, electron microscopy, and thermogravimetry, was previously reported.^54,55^ Dimethylformamide (DMF) (99.8%) purchased from Sigma-Aldrich was used as solvent to dissolve PCL and disperse GNP, being one of the best solvent for graphene and its related materials.^24^

### Preparation Methods

**Scheme 1:**
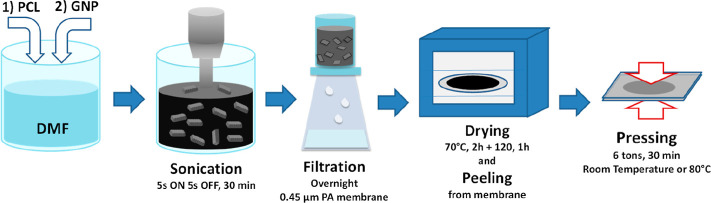
Preparation Procedure of the Nanopapers

Nanopapers were prepared by filtration following the procedure, presented in Scheme 1 and described hereunder.

Different amounts of PCL pellets (25, 50, 250 and 500 mg) were dissolved in 150 mL of DMF at 60 °C for 1 h to obtain solutions with different polymer concentrations. GNP powder (50 mg) was added to the prepared PCL solutions. Homogeneous suspensions (with no obvious big GNP particles when transferred to the filter) were obtained by applying a previously validated^56^ sonication treatment in pulsed mode (5 s on and 5 s off) for 30 min with power set at 30% of the full output power (500 W), accomplished with an ultrasonication probe (Sonics Vibracell VC-505, Sonics & Materials Inc.) with a 13 mm diameter Ti-alloy tip. The suspension was transferred into a filtration system equipped with a polyamide (PA) supported membrane (0.45 μm nominal pore size, diameter 47 mm, Whatman) and left for filtration overnight. After filtration, the cake, containing GNP and adsorbed PCL over the membrane, was dried in two steps; first, at 70 °C, for 2 h to remove most of the solvent and later at 120 °C for 1 h to complete solvent removal. Drying in two steps was adopted to avoid cracking of the film, observed when drying in one step at 120 °C, due to the high solvent evaporation rate. Finally, nanopapers were obtained by uniaxial compression into an hydraulic press (6 tons load for 30 min) of the PCL–GNP cakes after being peeled off from the membrane at room temperature (RT). Alternatively, hot pressing (80 °C and then cooled down to 30 °C by water cooling of compression plates) was applied to further consolidate the nanopaper structure. In particular, hot pressing was applied to larger nanopapers, prepared using 90 mm membrane filters and using 200 mg of GNP suspended in 600 mL of DMF, while maintaining the same preparation procedure. Samples codes were defined by indicating the initial ratio of PCL and GNP in the suspensions before filtering, the dimension of the prepared nanopapers and the pressing method, namely hot-pressed 80 °C or cold-pressed (RT), as shown in Table 1. Selected nanopapers were washed in Soxhlet in toluene for 12 h, to extract PCL, prior to further characterization.

**Table 1:** List of Prepared Nanopapers and Their Preparation Conditions

sample code	wt % ratio, PCL:GNP in suspension	filter diameter [mm]	pressing temperature
PCL10-GNP1	10:1	47	RT
PCL10-GNP1-H	10:1	90	80 °C
PCL5-GNP1	5:1	47	RT
PCL5-GNP1-H	5:1	90	80 °C
PCL1-GNP1	1:1	47	RT
PCL1-GNP1-H	1:1	90	80 °C
PCL1-GNP2	1:2	47	RT

### Characterization

#### Scanning Electron Microscopy

Scanning electron microscopy (SEM) micrographs were acquired by an EVO 15 SEM (Zeiss, Germany) with a beam voltage of 20 kV. The micrographs were taken on the nanopapers cross-section, obtained by fragile fracture, after soaking in liquid nitrogen.

#### Thermal Gravimetrical Analysis

Thermogravimetric analysis (TGA) was performed with a Mettler-Toledo TGA 1 thermogravimetric analyzer. Samples with weight of 5–8 mg were heated from 35 to 900 °C under a nitrogen flow of 80 mL/min and then were kept at 900 °C for 20 min under oxygen at the same flow rate. TGA measurements were carried out at least five times for each nanopaper to get an average and representative value of PCL content and its experimental deviation.

#### Differential Scanning Calorimetric (DSC): Non-Isothermal DSC Scans

Differential scanning calorimetry (DSC) experiments were performed under a continuous nitrogen purge on a Mettler calorimetric apparatus, model DSC1 STARe/E system. The samples, having a mass between 2.5 and 6 mg, were first heated from −10 to 200 °C, then cooled down to −100 °C and finally heated to 200 °C again. A scanning rate of 10 °C/min was used on both heating and cooling. Reported enthalpy values were calculated on the actual PCL content in PCL/GNP nanopapers, measured by TGA on the same sample after DSC test. Experimental errors are estimated in the range of ±1% of the initial weight.

The crystallinity (*X*_c_) of PCL in different nanopapers was calculated by considering their real contents, Φ_PCL_, following the eq 1
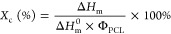
1where Δ*H*_m_ is the measured heat of fusion, with an estimated error of 10%, Φ_PCL_ is the PCL content in the nanopapers, and Δ*H*_m_^0^ is melting enthalpy of the 100% crystalline PCL (139.5 J/g).^57^

#### DSC: Successive Self-Nucleation and Annealing (SSA) Experiments

SSA experiments were performed with a PerkinElmer DSC 8500, connected to a liquid nitrogen cooling accessory (CLN2). The DSC was operated under a constant ultrapure nitrogen flow of 20 mL/min to maintain an inert atmosphere and calibrated with indium and zinc standards, using a scanning rate of 20 °C/min and a sample mass of 3 mg.

**Scheme 2:**
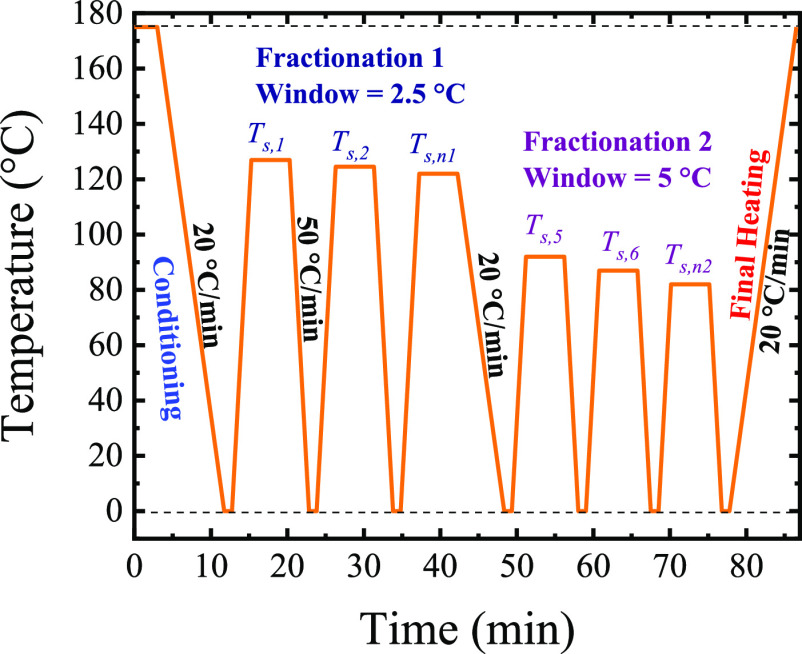
SSA Protocol Employed in All the Samples; The Holding Time at Each Self-Nucleation Temperature (*T*_s_) is 5 min; The Fractionation Window and Cooling and Heating Rates are Indicated

The SSA experiments were performed using the method created and reviewed by Müller et al.^58−61^ In this work, we used the SSA to explore the origin (through the study of the fractionation capacity) of high-temperature endothermic transitions, for example, 76, 85, and 120 °C. Therefore, we modified the standard SSA protocol because the mentioned transitions are much higher than the “ideal self-nucleation temperature”, *T*_s,ideal_, of the PCL. The employed protocol is schematically represented in Scheme 2 and described below:

(a) Conditioning: In this step, a standard thermal history was created. Therefore, the thermal history and crystalline memory were erased by heating up the sample to 175 °C and holding it at this temperature for 3 min. Then, the sample was cooled from the melt to 0 °C a 20 °C/min, creating the standard thermal history.
(b) Fractionation 1: To explore the highest endothermic peaks observed during the non-isothermal scans, we heated the sample until a self-nucleation temperature (*T*_s_) equal to the end melting temperature (*T*_m,end_) of the studied transition was achieved, *T*_s,1_–*T*_m,end_, for example, 127 °C, and held this temperature for 5 min. The sample was then cooled to 0 °C at 50 °C/min, held 1 min at 0 °C, and subsequently heated to the next *T*_s,2_ = *T*_s,1_ – 2.5 °C. To cover the highest melting peak, we used at least three *T*_s_ (e.g., 125, 122.5, and 120 °C) and employed a fractionation window of 2.5 °C.
(c) Fractionation 2: After the final *T*_s_ of fractionation 1, we cooled the sample to 0 °C at 20 °C/min, and the following heating scan was performed until a *T*_s_ adapted to the new endothermic peak to explore, for example, 92 °C. From this point onward, we select fractionation windows of 5 °C (also a thermal fractionation window of 2.5° was used in selected samples for comparison purposes) due to the broadness of these new endothermic peaks. In general, we covered a range of 50 °C (i.e., 92 to 42 °C) to fractionate the endothermic peaks observed at 85 and 76 °C during the non-isothermal tests. This protocol aims to fractionate the endothermic peaks at high temperatures and part of the PCL. The total fractionation of the PCL requires lower *T*_s_ values.
(d) Final Heating: After the final *T*_s_ of fractionation 2, we cooled down the sample to 0 °C, and subsequently heat the sample to 175 °C, at 20 °C/min, recording the different fractions generated in (b) and (c).

#### Structural Characterization: Wide-Angle X-ray Scattering (WAXS)

The structural characterization was performed in the 1W2A beamline of the Beijing Synchrotron radiation Facility (BSRF). Selected samples were previously fractionated in a Linkam THMS600 hot stage connected to a liquid nitrogen station. The final heating was performed from 25 to 140 °C at 5 °C/min in a Linkam TST350 hot stage, and the WAXS patterns were taken simultaneously. An exposure time of 25 s was used, and the patterns were taken every 30 s (i.e., every 2.5 °C). A Pilatus 1M detector collected the scattering patterns with a resolution of 981 × 1043 pixels (pixel size = 79 × 79 μm^2^). The sample-to-detector distance was 121.8 mm, and the wavelength was 1.54 Å.

WAXS experiments, on selected samples, were performed with transmission geometry at RT. These experiments were performed on a Xeuss 2.0 system (Xenocs SA), equipped with a microfocus Cu Kα X-ray source (GeniX3D, 50 kV, 0.6 mA), generating X-ray radiation of a wavelength of 1.54 Å. The detector used was a Pilatus 300K (DECTRIS, Swiss) with a resolution of 487 × 619 pixels (pixel size = 172 × 172 μm^2^). The sample-to-detector distance was 138.61 mm, and exposure time of 1800 s. The 1D intensity profiles were integrated from background-corrected 2D WAXS patterns with an azimuthal angle range of 0–90°.

### Thermomechanical Properties

Thermomechanical properties of nanopapers at different temperatures were investigated by using a Q800 dynamic mechanical analyzer by TA Instruments, in tensile mode, using 5 × 20 mm^2^ specimens cut from nanopapers. Tests during a heating ramp were carried out from RT to 150 °C at a heating rate of 2 °C/min, strain of 0.05%, and frequency of 1 Hz. Deformation under constant load tests (referred to as creep tests) were carried out at 120 °C under 5 MPa, for 8 h, followed by deformation recovery at zero load at the same temperature for 8 h.

### Thermal Diffusivity and Conductivity

The thermal diffusivity (α) of the prepared nanopapers was measured at 25 °C using the xenon light flash analysis (LFA, Netzsch 467 Hyperflash). The samples were cut in disks with a diameter of 25 mm, and the measurements were carried out in the in-plane sample holder, in which the sample is heated in the central region, and the temperature rise was measured on the outer ring of the sample. Measurements were carried out five times for each sample to get an average thermal diffusivity and experimental deviation.

Thermal conductivity was calculated from the measured diffusivity values, multiplied by the density and specific heat capacity of the different materials
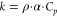
2where *k* is the thermal conductivity; ρ is the density of the nanopaper; *C*_*p*_ is the specific heat capacity of material.

The specific heat capacity of nanopapers (*C*_*p*n_) were calculated by the weighted average of *C*_*p*_ values of PCL (2.0 J g^–1^ K^–1^ at RT)^62^ and graphite (0.71 J g^–1^ K^–1^ at RT)^63^ for each sample
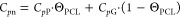
3where *C*_*p*P_ is the specific heat capacity of PCL; Θ_PCL_ is the weight percentage of PCL in the nanopapers; *C*_*p*G_ is the specific heat capacity of graphite.

## Results and Discussion

### Morphology of PCL/GNP Nanopapers

Composite nanopapers easily obtained by filtration of PCL/GNP suspension demonstrated high flexibility. Indeed, freestanding nanopapers can easily be bent and folded, then again restored to a planar shape, without breaking, which is not the case for the neat GNP nanopaper. Photographs of the pristine GNP and PCL10-GNP1 nanopapers after deformation are reported in Figure S1.

**Figure 1:**
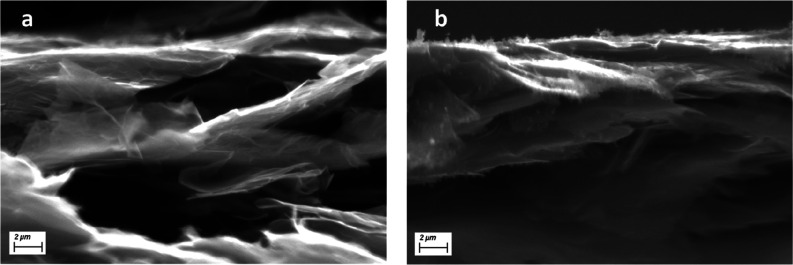
SEM micrographs for cross-section of GNP nanopaper (a) and PCL10-GNP1-H (b) taken as an example of PCL-containing nanopapers. In the latter, PCL is visible as a smooth-appearance phase covering the sharply defined of GNP flakes visible in pristine GNP nanopaper.

The morphology of nanopapers in cross-section was investigated by SEM (Figure 1), showing strong orientation as a consequence of filtration from suspension of nanoflakes, accordingly to our previous report.^56^ Furthermore, the presence of an additional phase is observed in composite nanopapers, which is more clearly visible in nanopapers prepared with higher PCL/GNP ratios, suggesting a higher PCL content in the obtained materials. Qualitatively, PCL appears strongly adhered to GNP (Figure 1b) and likely intercalated between thin galleries observed in pristine GNP nanopaper (Figure 1a).

Comparing PCL/GNP nanopapers compressed at RT (cold-pressed) versus the corresponding prepared by hot-pressing (Figures S2 and S3, respectively), significant differences can be found in both thickness and porosity. Indeed, RT compressed nanopapers (Figure S2) exhibit a higher thickness, typically in the range of 100 μm and a delaminated structure. On the other hand, hot-pressed counterparts (Figure S3) are clearly thinner (approx. 30–40 μm) and more compact, especially for higher PCL/GNP ratio, evidencing the hot-pressing stage to consolidate the structure once PCL is above its melting temperature.

### PCL Content Determination by Thermogravimetry

The amount of PCL retained by GNP flakes during filtration was investigated by thermogravimetry measurements (Figure S4). Because PCL has a much lower decomposition temperature (*T*_max_ at ca. 400 °C) than GNP, it is possible to calculate the polymer content inside the nanopapers from the residual weight at 600 °C, as reported in Table 2. The polymer fraction in the nanopapers is clearly much lower than the polymer concentration in the suspension, relative to GNP, demonstrating that only a limited fraction of PCL can be adsorbed onto the GNP flakes and retained in the nanopapers. However, the PCL concentration within the nanopapers can be increased by increasing the initial concentration of PCL in the suspensions, relative to GNP. Indeed, ca. 6 wt % PCL was obtained in PCL1-GNP2, whereas concentrations up to about 20 wt % were obtained for PCL10-GNP1-H. The PCL content in nanopapers is affected by the initial concentration of the polymers, but it appears to be mainly dependent on the interaction between PCL chains and GNP surface. When the concentration of PCL in the initial suspensions is low, such as PCL1-GNP1 and PCL1-GNP2, the low viscosity (qualitatively observed) of the PCL solution leads to a relatively fast filtration process. When the concentration of PCL solution is gradually increased, the viscosity is qualitatively observed to increase, which may contribute to retaining a higher PCL fraction.

**Table 2:** Actual PCL Content Determined by TGA, Enthalpies (Normalized on the Actual PCL Content) for Endothermic Transitions on Second Heating in DSC, and Crystallinity (*X*_c_) Calculated Considering the Total Enthalpy (Sum of Peaks Contributions)^a^

		Δ*H* (J/g_PCL_) of the peaks from 2nd heating scan					
sample	PCL content (wt %)	A	B	C	D	total	crystallinity, *X*_c_ (%)
neat PCL	100	66.3				66.3	47.5
PCL10-GNP1-H	20 ± 3	33.0	2.3	0.5		35.8	25.6
PCL10-GNP1	17 ± 3	27.2	2.8	0.9	0.8	31.7	22.7
PCL5-GNP1-H	15 ± 3	23.4	3.4	0.8		27.6	19.8
PCL5-GNP1	10 ± 1	24.0	3.6	1.0	1.2	29.8	21.3
PCL1-GNP1-H	7.6 ± 1	5.3	1.5	1.4	0.4	8.6	6.1
PCL1-GNP1	6.3 ± 0.5	4.5	2.7	1.9	4.1	13.2	9.4
PCL1-GNP2	6.0 ± 0.6	2.0	3.0	1.8	4.8	11.6	8.3

a A, B, C and D refers to peaks identified in Figure 2b.

### Non-Isothermal DSC Experiments

To investigate the organization of PCL chains between GNP, the PCL crystallinity within the nanopapers (Table 2), was addressed and found to strongly decrease in nanopapers, which is expected as confinement is known to decrease crystallinity.^64−67^ Besides the fundamental study, polymer crystallinity is also related to the envisaged application of these nanopapers in conductive films. Indeed, crystalline polymers exhibit higher thermal conductivity than amorphous polymers^38,40,42,43^ due to the ordered crystal structure. In contrast, the random chain conformation in amorphous polymers reduces the phonon mean free path and causes phonon scattering, thus decreasing the heat transfer efficiency.^39,43^

The crystallization and melting behaviors of the prepared nanopapers and the neat PCL were characterized by DSC, and the results are reported in Figures 2 and S6. During DSC cooling scans (Figure 2a), the crystallization of pristine PCL can be clearly observed as a sharp crystallization exothermic peak (*T*_c_) with a maximum temperature at ca. 28 °C, which is consistent with the previous *T*_c_ values of the of linear PCL.^68^ On the other hand, the *T*_c_ for PCL in the presence of GNP increased to ca. 47 °C, that is, about 20 °C higher than that of the neat PCL, suggesting a significant nucleation activity of GNP flakes on PCL. This crystallization peak is clearly visible for both PCL10-GNP1 and PCL10-GNP1-H, while significantly lower and broader signals were obtained for PCL5-GNP1, PCL1-GNP and PCL1-GNP2, which can be partially explained in terms of lower polymer contents (Table 2) within the latter nanopapers.

**Figure 2:**
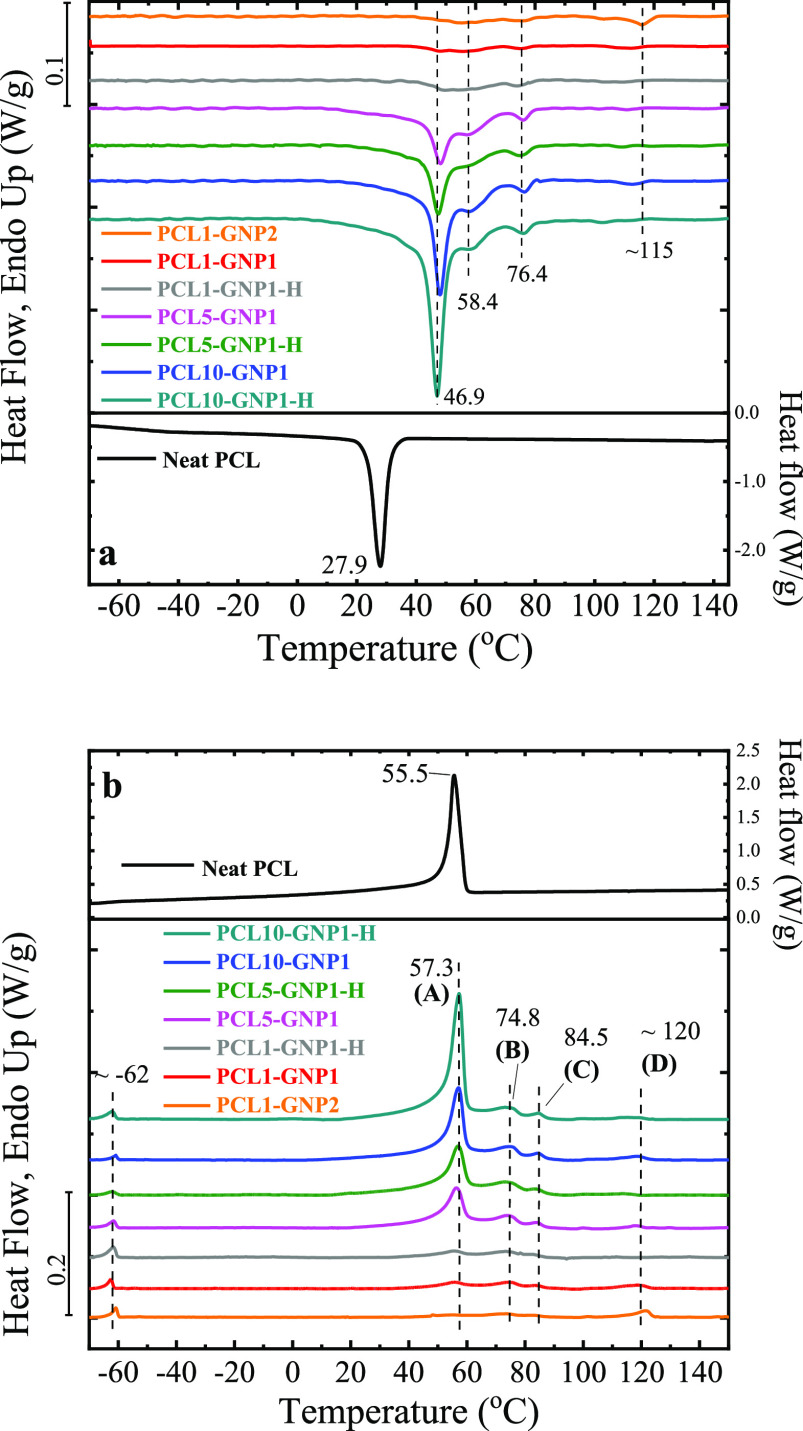
DSC curves for the cooling (a) and second heating (b) stage. In Figure 2b, the endothermic peaks are identify as A, B, C, and D.

The increased *T*_c_ for PCL within the nanopapers can be interpreted based on previous literature reports describing strong nucleation activity of GRM in nanocomposites.^69−72^ For PCL, Ahmed et al.^73^ reported the effect of GO on the non-isothermal crystallization behavior of PCL, demonstrating an increase in *T*_c_ of PCL/GO nanocomposite to ca. 35 °C, compared to ca. 26 °C for the neat PCL, with 1.0 wt % GO loading. Similar results were reported for a PCL/rGO nanocomposite by Wang et al.,^71^ with an increase of ca. 10 °C on *T*_c_ for the nanocomposite compared to neat PCL. Zhang and co-workers^74^ produced nanocomposites based on PCL and thermally reduced GO (TrGO) and reported that the *T*_c_ of the nanocomposite increased to around 36 °C with TrGO loading of 2 wt % from 25 °C for neat PCL. Zeng et al.^75^ studied the crystallization behavior of PCL/poly(sodium 4-styrenesulfonate) functionalized GNP (FGNP) composites. Under a cooling rate of 10 °C/min, they found that *T*_c_ increased ca. 8 and 11 °C with the addition of 0.05 and 1 wt % of FGNP, respectively. A detailed study of the non-isothermal crystallization behavior of PCL nanocomposites with graphite oxide and different loadings compared to pristine graphite powder was reported by Kai el at.,^76^ showing an increase in *T*_c_ for all prepared composites, within 10 °C. The crystallization temperature shifts obtained in this work are significantly higher than those previously reported for PCL-containing graphene-related materials, which can be explained by the limited fraction of PCL into the nanopapers, leading to a high interfacial area between GNP and the polymer chains, maximizing nucleation density.

Besides the *T*_c_ shifts, it is important to note that extra exothermic peaks at ca. 58, 76 °C and a broad signal above 100 °C were found for all the nanopapers, which are not present in neat PCL. Relative intensities for these signals, compared to the main crystallization peak, seem to increase when decreasing the total PCL content, thus suggesting such signals to become more important when having small amounts of PCL, strongly confined onto or between GNP flakes.

From the results of the second DSC heating scans, a main endothermic signal in the range between 55 and 58 °C (peak A), corresponding to the well-known melting of PCL, is clearly observable for both pristine polymers and nanopapers, except for PCL1-GNP2 (Figure 2b). Furthermore, additional signals are found in the DSC traces for the nanopapers. Indeed, a first distinctive features for the nanopapers is found at ca. −62 °C, which is assigned to the glass transition of PCL.^77^ This signal is not visible in pristine PCL, and may therefore suggest that a significant fraction of PCL in the nanopapers remains amorphous during the cooling stage.

It is worth noting that the peak melting point (*T*_m_) for PCL in the nanopapers is slightly higher in comparison to pristine PCL (i.e., 1.8 °C higher), a result that can be expected due to the much higher crystallization temperature of the PCL in the nanopapers (due to the graphene nucleating effect).

In addition, extra endothermic peaks at ca. 75 (peak B), 84 (peak C) and a broad signal around 120 °C (peak D) were observed for the composite nanopapers, which were not found for neat PCL (peak A), and corresponding to the above-described signals for the cooling stage, suggesting the existence of crystals with different PCL chain organization. Similar signals were also found on the first heating scans (Figure S6). To the best of the authors’ knowledge, this set of high-temperature endothermic transitions has never been reported for the crystallization of PCL, in a tridimensional structure, and are most likely related to a peculiar organization of PCL chains on the surface of GNP. Here, we study such organization through DSC and X-rays characterization, whereas the specific details of the interactions between the PCL and GNP surface are outside the scope of the present contribution.

In principle, melting peaks at higher temperatures may be related to higher stability PCL crystals, possibly characterized by thicker lamellae or different crystalline forms. However, the equilibrium melting temperature (*T*_m_^0^) of PCL has been reported to be, in most of the cases, in a range between 59.8 to 80 °C.^78^ Only few works have reported *T*_m_^0^ values at temperatures as high as 80 and 98 °C.^79−81^ It is expected that experimentally determined *T*_m_ values should be well below *T*_m_^0^.

Recently, *T*_m_ values as high as 80 and 84 °C have been reported by Thurn-Albrecht et al.^82,83^ They prepared thin PCL films (by solution in toluene, and then using spin coating on the selected surface) on freshly cleaved highly oriented pyrolytic graphite^82^ (HOPG) or onto molybdenum disulfide^83^ (MoS_2_), and then, by atomic force microscopy measurements, they detected that the PCL is totally molten only at 84 °C (HOPG) and 80 °C (MoS_2_). This behavior was attributed to the epitaxial crystallization of the PCL on the HOPG induced by pre-freezing. Pre-freezing is a first-order surface transition, and it is reversible (although certain hysteresis might exist). It is expected for strongly attractive surfaces or epitaxial systems (i.e., matching between the substrate’s lattice and crystalline materials).^84^ The pre-freezing phenomena can be understood as “the formation of a crystalline prewetting layer occurring under equilibrium conditions above *T*_m_”.^82^ The formation of the pre-frozen layer depends on the interfacial energy differences between substrate, melt, and crystals.^85^

**Figure 3:**
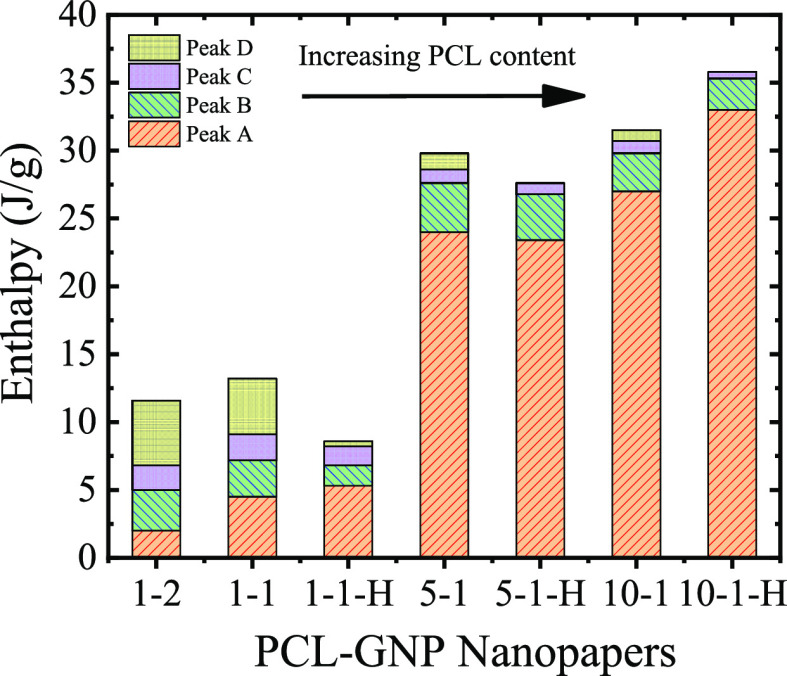
Integral enthalpy values of the endothermic peaks obtained during the 2nd DSC heating scans for the different nanopapers, as a function of the PCL content.

Based on the mentioned PCL literature, the observed endothermic peaks in this work at approx. 75 (peak B), 84 (peak C), and 120 °C (peak D) remain extremely unusual for PCL and their origin was investigated in detail. To quantify the relative amounts of the different crystalline populations, the enthalpies of the peaks obtained during the second DSC heating scans were calculated, taking into account the actual PCL contents in the nanopapers, and reported in Table 2. The melting enthalpy of the most intense peak [at ca. 57 °C (peak A)] was found to increase with the increase of PCL content in the nanopapers, whereas the opposite trend was found for peaks at higher temperatures (peak B, C, D) (Figure 3).

Trends for DSC signals suggest a strong role of GNP in organizing PCL crystals upon cooling. When a limited amount of PCL is present in between GNPs, the interaction between PCL chains and GNPs could promote the nucleation process. However, GNPs could also restrict cooperative movements of PCL chains, causing a reduction in the total crystallinity of PCL inside the nanopapers. Indeed, the total enthalpy, obtained as the sum of all the peaks for PCL in nanopapers and normalized by the actual PCL amount, is always lower than in pristine PCL and is found to decrease with decreasing PCL contents (see Table 2).

Hot pressing of nanopapers appears to have limited effect on the crystallization behavior of the PCL because the DSC profile of the hot-pressed versus cold-pressed nanopapers are similar (Figure 2), except for the high temperature endothermic peaks. Peak D is not clearly visible (PCL5-GNP1-H and PCL10-GNP1-H) or shows a lower (PCL1-GNP1-H) enthalpy (see Figure 3 and Table 2) in hot-pressed samples, but peak D is not only related to cold-pressed samples because it also appears in unpressed ones (see Figure S7). On the other hand, the differences in enthalpies for the other peaks (A to C) in hot-pressed versus cold-pressed nanopapers are comparable, taking into account the experimental errors.

Aiming at elucidating the interactions between PCL and GNP within the nanopapers, selected nanopapers were thoroughly washed in toluene to extract unbound PCL. After extraction, both PCL10-GNP1 and PCL1-GNP1 exhibited equivalent PCL contents in the range of 3 to 4 wt %, which represents a significant reduction from the 17 ± 3 and 6.3 ± 0.5 wt %, respectively, detected in as prepared nanopapers (Table 2 and Figure S5). These results evidence that a limited amount of PCL can either strongly adsorb onto GNPs, which does not allow for extraction by toluene (although this is a well-known good solvent for PCL), or cannot be effectively reached by the toluene due to the fact that it might be trapped in between graphite nanolayers or galleries, avoiding or limiting the extraction. Furthermore, the amount of adsorbed PCL appears to be approximately independent on the total PCL content in as-prepared nanopapers, suggesting that the bound PCL fraction is limited by the surface area of GNP.

**Figure 4:**
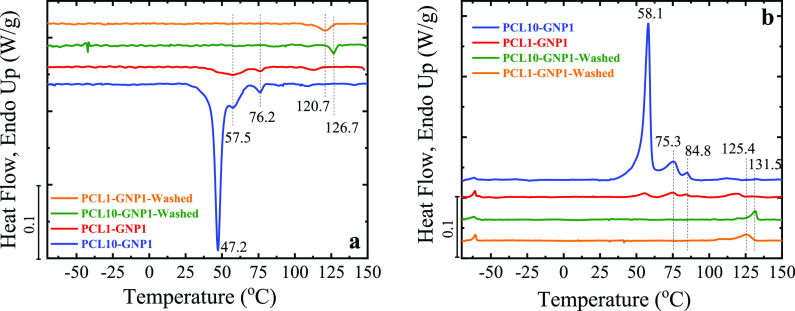
DSC curves for the cooling (a) and second heating (b) stage for selected nanopapers (i.e., PCL1-GNP1 and PCL10-GNP1), before and after washing in toluene.

To correlate the presence of adsorbed PCL with the DSC signals discussed above, DSC measurements were also carried out on toluene-washed nanopapers, as reported in Figure 4. On both cooling and heating DSC scans, the lower temperature signals observed in as-obtained nanopapers disappear after washing. Although a strong reduction in the melting enthalpies is expected, accordingly with the PCL content reduction observed by TGA, it has to be noted that the only DSC signals detectable on washed nanopapers are at temperatures above 120 °C. Indeed, both PCL10-GNP1 and PCL1-GNP1 present corresponding signals on cooling (exo) and heating (endo), with a difference in temperature of approx. 5 °C, demonstrating a reversible transition. These signals appear to be related to the previously described high temperature transitions in as-obtained nanopapers. However, in washed nanopapers, the transition occurs at higher temperature and the signal is more intense, despite the reduction in PCL content. It is therefore apparent that the PCL fraction strongly adsorbed onto GNPs is responsible for the high-temperature transition (i.e., peak D).

### SSA Experiments

The SSA technique^58−61^ combines a series of non-isothermal (i.e., cooling and heating cycles) and isothermal steps (i.e., holding steps at each *T*_s_), designed to produce a series of thermal fractions with distinct melting peaks, that is, corresponding to a distribution of lamellar thickness. Such distribution is best created in semi-crystalline polymers where defects (e.g., chain branches, comonomers, or stereo-defects) interrupt the linear crystallizable chain sequences. Nevertheless, differences in molecular weight distribution or chain length can also generate fractionation. The fractionation or SSA profile is revealed in the final SSA DSC heating scan. The different peaks indicate the melting of crystallites with different lamellar thicknesses formed and annealed at each *T*_s_. In linear (defects free) polymeric chains, the fractionation capacity is low, for example in high density polyethylene.^58^ Still, some fractionation can be detected compared to non-isothermal tests. In that sense, if there is any crystalline material, the SSA test should generate changes in the melting point distribution within the sample.

In this work, we use the SSA experiments to study the origin (through the study or their fractionation capacity) of endothermic peaks at unexpected higher temperatures (i.e., higher than the reported *T*_m_^0^ of the PCL), detected during non-isothermal experiments (see Figures 2 and 4). The peaks at higher temperatures (e.g., peak D in Figure 2) are stronger in the cold-pressed samples. Therefore, we selected these samples for the SSA fractionation and X-ray characterization (see section WAXS Experiments).

**Figure 5:**
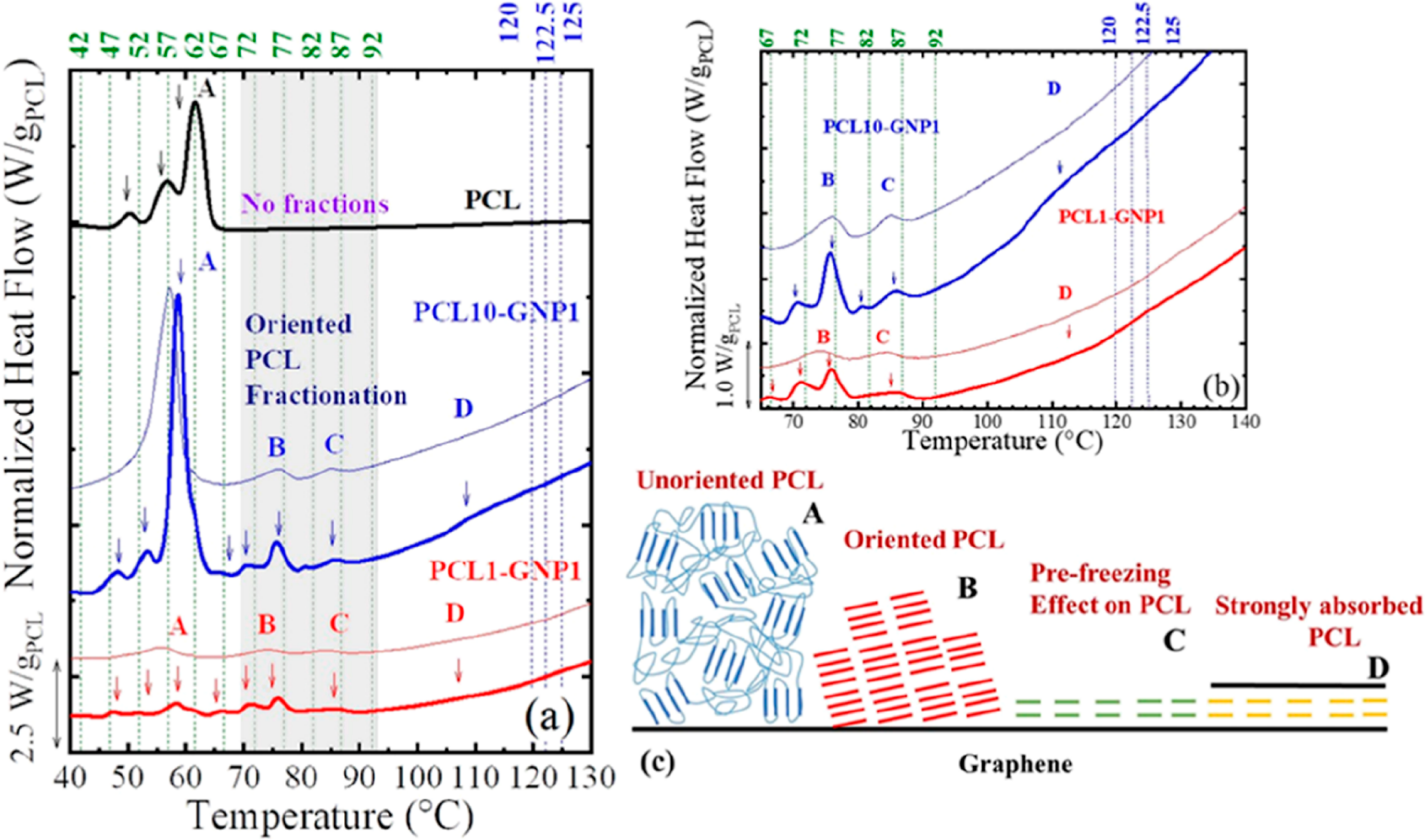
(a) SSA final heating for the neat PCL, and the PCL1-GNP1, and PCL10-GNP1. The blue dotted lines indicate the fractionation at high temperatures (fractionation 1), with fractionation windows of 2.5 °C. In contrast, the green dotted lines indicate the fractionation performed at lower temperatures (fractionation 2), with fractionation windows of 5 °C. The *T*_s_ values employed are displayed at the top of the figure. We used the letters A, B, C and D (employed in Figure 2) to differentiate the different fractions’ origin, and for clarity, we added the second heating DSC curves of unfractionated samples (plotted with thinner lines) of the nanopapers. (b) Close-up of the nanopapers SSA curves around peaks B to D, thinner lines are for unfractionated samples. (c) Illustration of the possible origin of peaks A to D. Note that the weight of PCL normalized all the curves in each sample.

Figure 5 compares the SSA final heating scans of neat PCL, PCL1-GNP1, and PCL10-GNP1. The SSA final heating of other samples is shown in Supporting Information, Figures S8–S10.

Figure 5a shows that the first fractionation protocol applied at high temperatures (i.e., fractionation 1, see experimental part) does not generate any apparent fractions (see the comparison of standard and fractionated heating curve in Figures 5 and S8–S10) in the already small endothermic peak D signal. For clarity, we have zoomed in (see Figure 5b) the curves around the peaks B to D for the nanopapers. This figure shows the unfractionated peak D, indicating that the PCL strongly absorbed onto GNPs (producing the endothermic signal at the highest possible temperatures), cannot be fractionated (due to its organization), as we described in the previous section.

For the second fractionation protocol, at lower temperatures (i.e., fractionation 2, see Experimental Section), the *T*_s_ values that encompass a endothermic peak C (∼85 °C) (see C in Figure 5a,b) do not generate fractions (see Figures S8–S12); hence, this peak remains unchanged, compared to the standard test (see the thinner lines corresponding to the second heating scan of the unfractionated samples in Figure 5a,b). A fractionation window of 2.5° was used to confirm that peak C remains unfractionated, independently of the fractionation window (see Figures S10 and S11). Thus, peak C should have a similar origin to peak D (∼125 °C).

In contrast, those *T*_s_ temperatures encompassing peak B (∼75 °C) (see B in Figure 5a,b) generate thermal fractions, indicating that its origin corresponds to PCL crystals that reach large lamellar thickness, induced by the GNP surface. The highest melting temperature fraction of peak B reaches ∼78 °C. This temperature is comparable to the upper limit of most of the reported *T*_m_^0^ values in the literature, that is, ∼59.8 to 80 °C.^78^ Only a few works have reported higher *T*_m_^0^ – 80–98 °C.^79−81^ For *T*_s_ < 67 °C (see A in Figure 5), the endothermic signal corresponding to usual PCL melting temperatures (peak A) is well fractionated. This result corroborates that melting peak A corresponds to the “standard” melting of PCL crystals (i.e., without a special organization induced by the GNP).

From the above-described results, we have detected that the endothermic transitions C and D (i.e., ∼85 and 125 °C) do not show significant fractionation due to their origin. Below (and in the illustration in Figure 5c), we speculate about the origin of peaks C and D.

The position of peak C and the absence of fractionation might be evidence of the pre-freezing phenomena. Thurn-Albrecht et al. also studied PCL on HOPG.^82^ They interestingly obtained that the pre-frozen layer melts at 84 °C, which is in line with our peak C. Therefore, the hypothesis of PCL pre-freezing effect on the surface of the low defectiveness GNP flakes^54,55^ (see Figure 5c) appears plausible to explain peak C.

Peak D, which is ∼40 °C higher than peak C, might have different origins. One possible explanation is the occurring of pre-freezing phenomena between graphite layers (galleries), as illustrated in Figure 5c. Compared to the pre-freezing onto a surface (HOPG or GNP), a corresponding phenomenon occurring within galleries between two GNP surfaces might increase the substrate–polymer interactions, increasing the *T*_m_. This is supported by reported results on pre-frozen polyethylene (PE) films indicating that substrate interactions play a crucial role in the pre-freezing phenomena. Indeed, MoS_2_, having stronger interactions with PE, causes an increase of the *T*_m_ (of the pre-frozen layer) higher than the one found for PE films in HOPG. The pre-frozen layer disappears at 155 and 124 °C for MoS_2_ and HOPG, respectively.^83,86^ Another possible explanation for peak D may involve a strongly adsorbed layer that might force the PCL crystals to crystallize in their extended chain conformation. For all these scenarios, the annealing of the PCL is indeed not expected, either because it is strongly bonded or because it is already in its extended conformation, explaining the absence of fractionation in peaks C and D.

It should be noted that the thermal history can affect the pre-freezing phenomena. Tournier and Ojovan^85^ found (through modelling) that the thermal history is more important than the substrate’s nature. They claim that pre-frozen layers are due to melt memory effects. Consequently, considering the thermal history influence, beside other factors, the study of the exact origin of peaks C and D is very complex and more studies are needed to establish their origin with absolute certainty.

Conversely, peaks B and A can be fractionated and correspond to oriented PCL on the GNP surface (∼76 °C) and unoriented PCL (<60 °C), respectively. Figure 5c illustrates four possible levels of PCL’s organization for each peak. The solid thick lines in Figure 5c represent the PCL crystals onto the GNP surface (solid black line), and the thinner lines represent the amorphous chains (only included in bulk PCL for simplicity). Crystals belonging to peak A are randomly distributed or unoriented on the GNP surface (hence they can be fractionated). Those crystals corresponding to the melting peak B have a certain level of orientation (note that they are not parallel to the GNP surface) and still can be fractionated. Concerning peak C, in which an epitaxial crystallization on GNP is likely occurring, the crystals appear to be parallel to the GNP surface and hence highly oriented due to the pre-freezing effect. Finally, we assume that the crystals corresponding to peak D are still highly oriented and particularly stable as a consequence of confinement between layers of GNPs.

The SSA final heating on peak B reflects different fractionation profiles depending on the PCL content, due to a confinement effect (see Figures S11 and S12). This effect is similar to the one found by Lorenzo et al.^87^ in PS-*b*-PE block copolymers. The PE block crystallizes within the microphase separated spaces dictated by the composition (lamellae, cylinders and spheres, respectively, as PE content decreases in the copolymers) in these strongly segregated copolymers. Thus, the highest thermal fraction is depleted, and lower fractions show a higher area instead. If we considered the partial areas of the endotherms of peak A and B, we find that as the ratio PCL/GNP decreases, the oriented PCL area (i.e., peak B) increases and the unoriented PCL one decreases (see Table S1 and Figures S8–S10), in line with the findings on non-isothermal tests (Figure 2).

### WAXS Experiments

**Figure 6:**
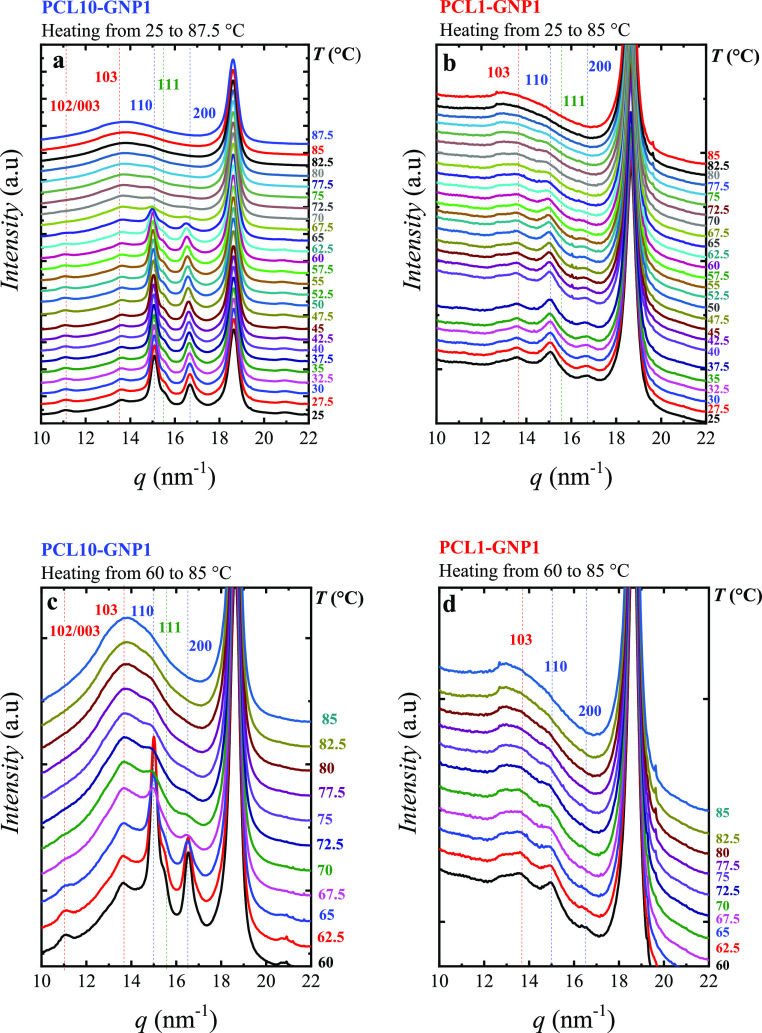
WAXS patterns taken during heating at the selected range for (a,c) PCL10-GNP1 at ranges of 25–87.5, and 60–85 °C, respectively, and (b,d) PCL1-GNP1 at ranges of 25–85, and 60–85 °C, respectively. The vertical dashed lines indicate the position of the PCL main planes.

The SSA technique allowed us to obtain information relating to the main endothermic transitions detected at high temperatures. The endothermic transitions correspond to the melting of unoriented PCL crystals (∼57 °C), oriented PCL crystals (∼78 °C), and to other processes (e.g., pre-freezing) of strongly interacting PCL chains on the GNP (∼85 and 125 °C). To understand these transitions, we performed WAXS experiments in previously fractionated samples (i.e., without the final heating), in the selected PCL1-GNP1 and PCL10-GNP1 nanopapers (see their SSA final heating in Figures 5 and S10). The SSA final heating was performed at 5 °C/min, and WAXS patterns were taken simultaneously. Figure 6a,b show the WAXS patterns taken, every 2.5 °C, during the SSA final heating of PCL10-GNP1 and PCL1-GNP1, respectively, in the range of 25 to ∼85 °C. In Figure 6c,d, we show only the range of 60 to 85 °C for both samples.

Figure 6 shows PCL crystalline reflections at *q* = 15 and 16.5 nm^–1^, corresponding to WAXS diffraction by the (110) and (200) crystal planes, and the characteristic shoulder at 15.5 nm^–1^, corresponding to the (111) reflection. These reflections are consistent with the PCL orthorhombic unit cell with *a* = 0.748, *b* = 0.498, and *c* = 1.726 nm.^88−90^ Interestingly, we also found weaker reflections, which are not typically reported in the literature, at *q* = 11 and 13.6 nm^–1^ that correspond to the (102)/(003) and (103) planes.^88−90^ The intense peak at *q* ∼ 19 nm^–1^ corresponds to the GNP in both PCL10-GNP1 (Figure 6a) and PCL1-GNP1 (Figure 6b). This signal can be indexed to the (002) reflections of the graphite.^91^ Upon heating, there is a shift to lower *q* values (i.e., higher *d*-spacing) of the PCL signals, resulting from the thermal expansion.

Figure 6b also shows that the peaks of the PCL1-GNP1 are weaker compared to the PCL10-GNP1 (Figure 6a), due to a lower crystallinity (see Table 2). Interestingly, the reflections of the (103) and (110) planes have comparable intensities. This might support a different arrangement in the PCL1-GNP1 nanopaper. In Figure 6c,d, we selected the WAXS patterns from 60 to 85 °C, which correspond to the oriented PCL (note that the unoriented PCL should be molten or in the end of its melting process). In both figures, it is observed how the main reflections of the PCL become less intense at 67.5 °C, indicating that the unoriented PCL is molten. With the unoriented PCL molten, the planes (103) and (110) generate the most intense reflections (and they also have comparable intensities, as in the PCL1-GNP1 sample), indicating a different crystalline arrangement related to the oriented PCL. From 77.5 to 80 °C, the signal of these planes, despite being weak, still prevailed. Hence, the oriented PCL crystals can melt at comparable melting temperatures to those reported as PCL equilibrium melting temperatures.^78^ The PCL1-GNP1 shows similar behavior.

For both PCL10-GNP1 and PCL1-GNP1, at 85 °C, all PCL crystalline reflections apparently disappear. Figure S13 shows the WAXS patterns taken from 87.5 to 130 °C for both PCL10-GNP1 and PCL1-GNP1, in which only the amorphous halo is detected for the PCL, together with the GNP corresponding signal. It is worth noting that at temperatures higher than 85 °C, the strongly absorbed PCL onto GNP does show endothermic transitions. As we mentioned before, peak C resembles the melting of a pre-frozen layer of PCL, similar to that reported by Thurn-Albrecht et al.^82^ They found that the PCL crystallizes in its (110) planes parallel to the graphite surface, whereas the (111) and (200) reflections are missing due to the epitaxial orientation.^82^ In Thurn-Albrecht et al. work, a whole monolayer of PCL crystallizes due to the pre-freezing phenomena. In contrast, we have different layers or organization levels in this work (see Figure 5c), hence different diffraction volumes. Considering the crystallinities related to each peak, the ones corresponding to peaks C and D are significantly lower. Thus, these peaks have a substantially lower diffraction volume, causing low signal intensity, which might be masked (overlapping of signals) by the amorphous halo (generated by peaks A and B with a higher diffraction volume). To avoid such a mask effect and increase the diffraction volume of peak D, we perform XRD experiments (Figure S14) in the washed PCL10-GNP1 (where only peak D was found by DSC), confirming the presence of small reflections generated by the PCL.

So far, the thermal and structural analysis indicated the presence of well-defined unoriented and oriented PCL. To proof the presence of the latter, we perform WAXS experiments under a transmission configuration. These experiments reveal the reflections of different planes (see Figure S15a), and a clear anisotropy (see inserted image on Figure S15b). Additionally, the azimuthal profile of the PCL10-GNP1 (see Figure S15b), evidences the orientation on the PCL, corroborating our previous findings.

### Thermomechanical Properties

**Figure 7:**
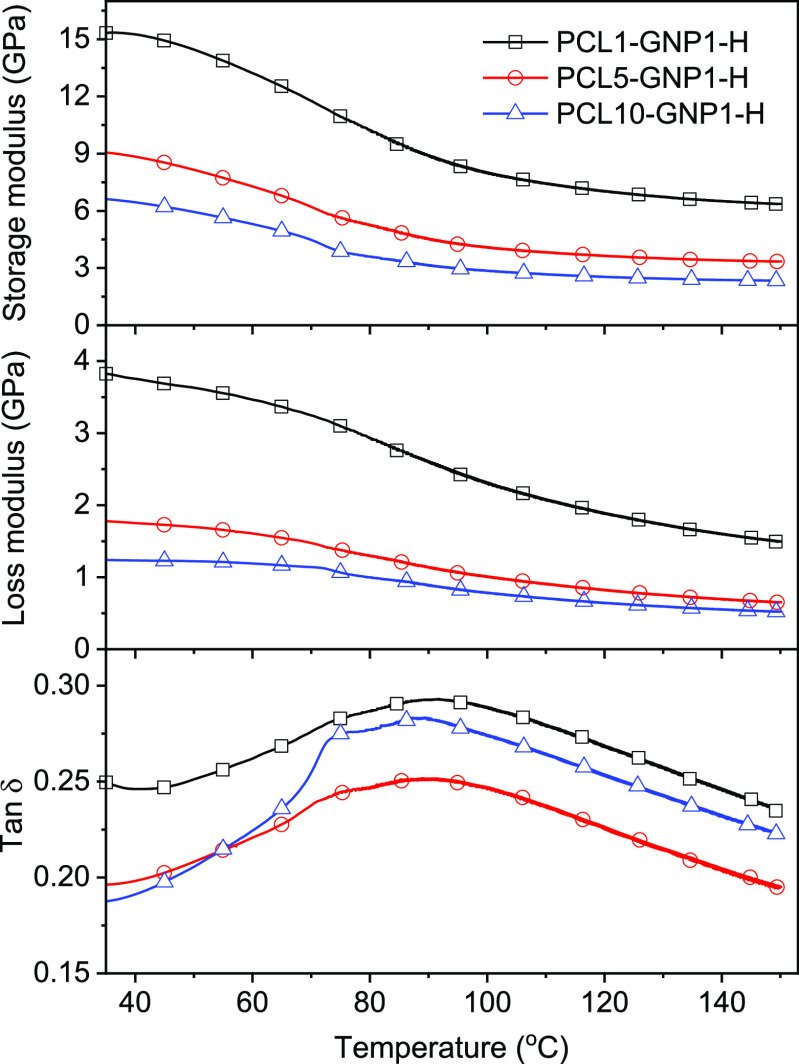
Temperature sweep DMTA measurement on selected nanopapers.

To investigate the thermomechanical properties of the nanopapers, dynamic-mechanical temperature sweep measurements were performed on hot-pressed nanopapers, namely, PCL10-GNP1-H, PCL5-GNP1-H, and PCL1-GNP1-H, taking into account their improved density and homogeneity, as revealed by SEM observations. These nanopapers demonstrated a significant stiffness at RT, with a storage modulus ranging between approx. 7 and 15 GPa, higher stiffness corresponding to lower PCL content, as expected. Interestingly, the storage and loss moduli decay versus temperature is relatively limited and remarkable stiffness is retained for temperatures far above the melting of PCL. Indeed, the storage modulus at 150 °C is about 2.3 and 6.4 GPa of PCL10-GNP1-H and PCL1-GNP1-H, respectively, suggesting a very strong adhesion of GNP plates to PCL, even after PCL melting. The *α* transition, taken as the maximum of tan δ, is observable at about 90 °C in all nanopapers (see Figure 7), suggesting a remarkable constraint of PCL macromolecules in the molten state, by the direct interaction with GNP or due to the highly stable pre-frozen PCL crystals.

The influence of PCL chains on the load-bearing capability of GNP nanopapers was further investigated by creep tests. Enhanced creep resistance was previously reported for various GRM polymer nanocomposites,^92−96^ showing that very low fractions of dispersed nanoflakes are able to reduce creep rate, which is generally explained by the formation of a percolating network of solid particles and/or by the constraint of macromolecules relaxation in the presence of dispersed particles. On the other hand, the creep of polymer-bound GRM nanopapers was not previously reported to the best of the authors’ knowledge.

**Figure 8:**
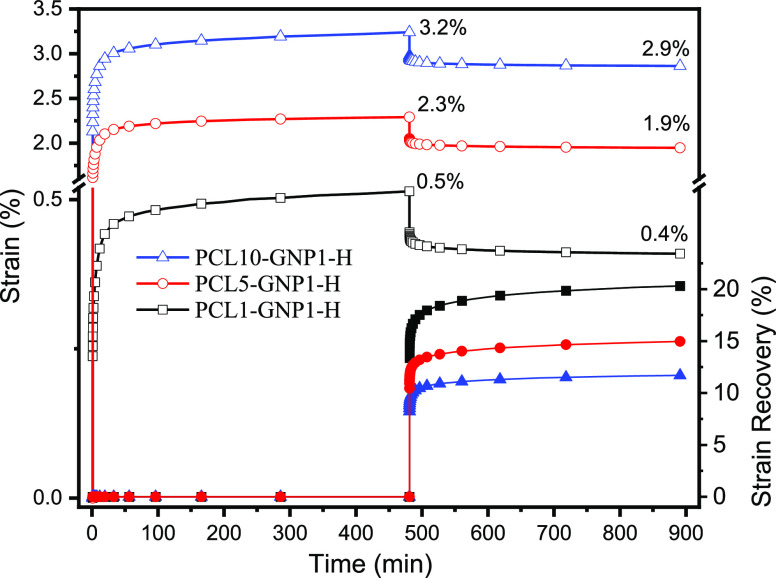
Strain (empty symbols) and strain recovery (filled symbols) plots from creep tests at 120 °C, 5 MPa stress on selected nanopapers.

Preliminary tests on our PCL/GNP nanopapers highlighted a negligible deformation at temperatures below the polymer melting point, a range in which creep is conventionally studied in polymers and polymer nanocomposites. Indeed, creep tests were carried out at 120 °C under 5 MPa stress, which is representative of the operating conditions of a low temperature heat exchanger, and the results are reported in Figure 8. Upon application of the constant stress, the PCL10-GNP1-H nanopaper immediately deformed to a strain of ca. 2.5%, followed by a further increase in strain, typical of phase I and II in creep tests, leading to a strain of 3.2% after 8 h creep at 120 °C. After the release of stress, the immediate strain recovery is around 9% of the strain after creep and the final value after 8 h recovery is close to 12% (i.e., a final deformation of 2.9%). As expected, creep resistance is even higher for PCL5-GNP1-H and PCL1-GNP1-H, owing to the lower PCL content, leading to 2.3 and 0.5% deformation after 8 h, respectively, which is partially recovered, leading to a final deformation of approx. 1.9 and 0.4%, respectively.

These results evidence the outstanding creep resistance of PCL/GNP nanopapers, at temperatures far above the melting of unoriented PCL. According to the above reported characterization of the highly stable PCL crystalline fractions (melting points up to above 120 °C), the remarkably high creep resistance is explained by the presence of crystalline PCL structures strongly bound to GNP flakes, directly connected to amorphous PCL fraction, thus acting as physical crosslinking points.

### Thermal Conductivity

Envisaging application of these flexible PCL/GNP nanopapers as heat spreaders, thermal diffusivity was measured and reported in Table 3. Pristine GNP nanopapers have a thermal diffusivity in the range of 150 mm^2^/s, which may be competitive with traditional metal foils.^56,97^ Diffusivity values for the GNP/PCL nanopapers are slightly reduced to 146 ± 2, 127 ± 1, and 138 ± 5 mm^2^/s for PCL1-GNP1-H, PCL5-GNP1-H, and PCL10-GNP1-H, according with the inclusion of a poorly conductive polymer.

**Table 3:** Thermal Diffusivity and Thermal Conductivity Values for Selected Nanopapers

sample	Φ_PCL_ [wt %]	density [g/cm^3^]	thermal diffusivity [mm^2^/s]	thermal conductivity [W/m·K]
GNP		1.0 ± 0.1	150 ± 2	106 ± 11
PCL10-GNP1-H	20 ± 3	1.3 ± 0.1	138 ± 5	175 ± 16
PCL5-GNP1-H	15 ± 3	1.4 ± 0.1	127 ± 2	160 ± 15
PCL1-GNP1-H	7.6 ± 1	1.4 ± 0.1	146 ± 2	191 ± 17

While thermal diffusivity represents the efficiency of heat spreading onto a surface, the heat flux obtained in a heat exchanger, given a certain temperature gradient, is quantified by the thermal conductivity. Thermal conductivity values are dependent on the product of thermal diffusivity and nanopaper density, is the latter being lowest for pristine GNP nanopaper and increased after hot pressing in the presence of PCL, thus compensating for the decrease in thermal diffusivity. Thanks to the existence of continuous GNPs networks and relatively high density, in-plane thermal conductivity yields outstanding values in the range of 160–190 W/m·K, which are much higher than that of reported for conventional GRM nanocomposites, embedding limited nanoparticles concentrations.^55,98−101^

The nanopapers prepared in this work target superior thermal conductivity properties coupled with high thermomechanical properties, which effectively bridge the property domains of polymeric materials and conductive ceramics. A comparison between our material and state of the art highly filled polymer composites is reported in Table 4, demonstrating that the best compromise between thermal conductivity and stiffness was reached. This excellent result was obtained, thanks to the combination of thermally conducive GNP and PCL. The PCL adds both high flexibility, due to its highly mobile amorphous chains, and physical cross-linking granted by the presence of a highly stable crystalline fraction that strongly interacts with the GNP surface. In particular, materials reported in this work are competitive with nanopapers based on the combination of graphene related materials and nanocellulose fibers,^102−106^ the latter providing a significant contribution to both stiffness and thermal conductivity of the nanopaper, thanks to their high crystallinity, strength and aspect ratio.

**Table 4:** Performance Comparison Between Nanopapers in This Work and Selected State of the Art Thermally Conductive Polymer Composites Prepared with High Loadings of Thermally Conductive Particles

material	conductive particles loading	thermal conductivity (W/m·K)	elastic modulus (GPa)	references
liquid crystal polymer/graphite	70 wt %	28.3	15	(107)
polybenzoxazine/boron nitride	78 vol %	32.5	10	(108)
polydimethylsiloxane/vertically aligned graphene film	92 wt %	615	0.5	(109)
cellulose nanofibers/graphene	50 wt %	165	2.6	(102)
cellulose nanofibers/graphene	90 wt %	240	2.0	(103)
cellulose nanofibers/rGO	50 wt %	7.3	7.5	(104)
cellulose nanofibers/GNP	75 wt %	59.5	5.0	(105)
cellulose nanofibers/PE oxide/expanded graphite	92 wt %	302	8.8	(106)
PCL/GNP	92 wt %	**191**	**15**	**this work**

## Conclusions

In this paper, PCL was successfully exploited as a polymer binder to enhance thermomechanical properties of GNP nanopapers. The organization of PCL within the highly oriented structure of the nanopapers was studied in detail. The crystallinity of PCL was found to be dramatically affected by GNPs, inducing a fascinating set of crystalline features. The amount of conventional and randomly oriented PCL crystals, with a melting temperature (approx. 57 °C) comparable to pristine PCL, was found to depend on the total PCL content. In particular, this crystalline population is dominant in nanopapers with higher PCL content and becomes progressively less important when reducing the polymer content. Besides, other crystalline populations increase in their relative ratio at low PCL content, suggesting organization of macromolecules to be strongly affected by the GNP. Indeed, oriented PCL crystals are found as a consequence of the strong nucleation effect, leading to higher stability crystals, corresponding to melting temperature in the range of 75 °C. Furthermore, additional endothermic transitions were found on heating at higher temperatures (approx. 85 and 120 °C), corresponding to fully reversible first order transitions of PCL. The nature of these transitions, occurring above the equilibrium melting temperature for PCL, was investigated and explained by the formation of crystalline pre-wetting layers, which can be in equilibrium above the melting temperature. The presence of high thermal stability crystals, strongly bound to GNP flakes, coexisting with the highly flexible amorphous structure, delivers an ideal solution for the toughening of GNP nanopapers. In fact, thermomechanical properties of PCL/GNP nanopapers demonstrated superior stiffness (ca 6 to 15 GPa, depending on the PCL content) at RT, which is mostly retained up to 150 °C. This property delivers excellent creep-resistance performance at 120 °C, which is required for components operating under high temperature and continuous mechanical stress. Most importantly, PCL/GNP nanopapers delivered thermal conductivities above 150 W/m·K, which makes those competitive with traditional metals in terms of heat dissipation, while granting for flexibility and lightweight, which cannot be matched by conventional thermally conductive metals or ceramics.

Based on the unique set of properties obtained for PCL/GNP nanopapers presented in this work, applications are envisaged in the field of flexible heat spreader devices, including but not limited to flexible electronics and low temperature heat recovery, and in wearable and implantable electronic devices. The demonstrated possibility to form polymer crystals stable above the equilibrium melting temperature may also have a broader impact in the field of nanostructures based on graphene and related materials, by the self-assembly of highly ordered hierarchical nanostructures with different populations of crystals of increasing thermodynamic stability.

## Supporting Information Available

Images of bent nanopapers, SEM images, TGA analyses, additional non-isothermal DSC scans and SSA results, additional WAXS, including orientation analysis, and XRD results (PDF)
