# Supplementary material for: Polycaprolactone Adsorption and Nucleation onto Graphite Nanoplates for Highly Flexible, Thermally Conductive, and Thermomechanically Stiff Nanopapers
Source: ACS Appl Mater Interfaces. 2021 Dec 1;13(49):59206–20. doi: 10.1021/acsami.1c16201 (PMC8678991; doi:10.1021/acsami.1c16201)
Supplement: Supplementary file 1 — am1c16201_si_001.pdf [file am1c16201_si_001.pdf]

SUPPLEMENTARY INFORMATION

Polycaprolactone adsorption and nucleation onto graphite nanoplates for highly flexible, thermally conductive and thermomechanically stiff nanopapers

Kun Li[1], Daniele Battegazzore[2], Ricardo A. Pérez-Camargo[3], Guoming Liu[3,4], Orietta Monticelli[1], Alejandro J. Müller*[5,6] and Alberto Fina*[2]

[1] Dipartimento di Chimica e Chimica Industriale, Università di Genova, Via Dodecaneso 31, 16146 Genova, Italy
[2] Dipartimento di Scienza Applicata e Tecnologia, Politecnico di Torino- Alessandria campus, viale Teresa Michel, 5, 15121 Alessandria, Italy
[3] Beijing National Laboratory for Molecular Sciences, Institute of Chemistry, Chinese Academy of Sciences, Beijing 100190, China
[4] University of Chinese Academy of Sciences, Beijing 100049, China
[5] POLYMAT and Department of Polymers and Advanced Materials: Physics, Chemistry and Technology, Faculty of Chemistry, University of the Basque Country UPV/EHU, Paseo Manuel de Lardizabal 3, 20018, Donostia-San Sebastián, Spain
[6] IKERBASQUE, Basque Foundation for Science, Bilbao, 48009, Spain
*Corresponding authors: alejandrojesus.muller@ehu.es; alberto.fina@polito.it

Section S1. Flexibility of Nanopapers

Flexibility and resistance to cracking under deformation was qualitatively evaluated by carefully controlled manual bending of the films. Images taken after bending and folding are reported in Figure S1. No visible cracks are visually observed in PCL10-GNP1 nanopapers after bending and folding, allowing recovery of the initial shape. Conversely, cracks are clearly observed in pristine GNP nanopaper (Figure S1 f, h), providing a qualitative indication of the material brittleness.

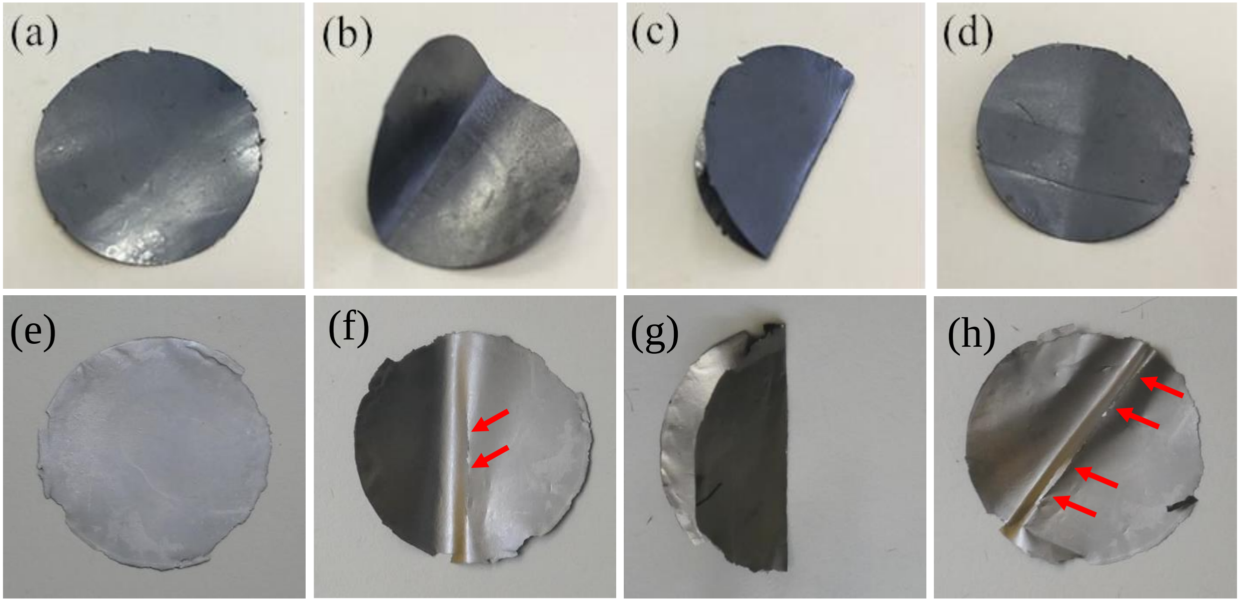

Figure S1. Photographs of freestanding PCL10-GNP1 (a-d) and pristine GNP nanopaper (e-h): initial nanopapers (a, e); nanopapers after 90° bending (b, f); folded nanopapers (c, g); recovery after being bent and folded (d, h). Red arrows indicate cracks after bending/folding of the pristine GNP nanopaper.

Section S2. SEM characterization

	Low Magnification	High Magnification
**GNP**	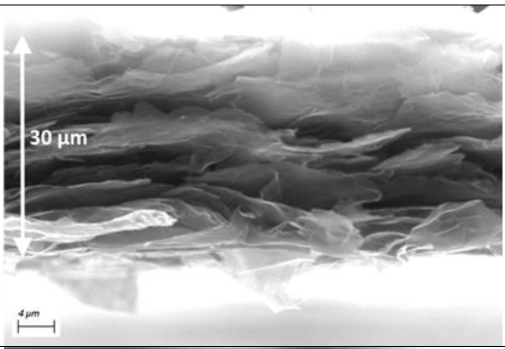	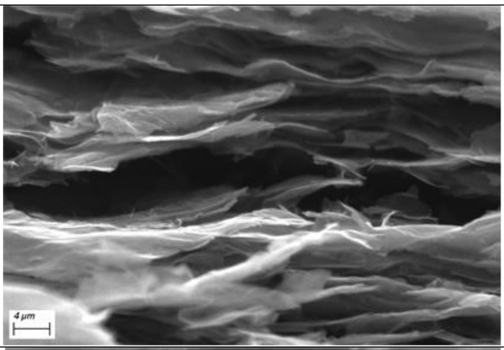
**PCL1-GNP2**	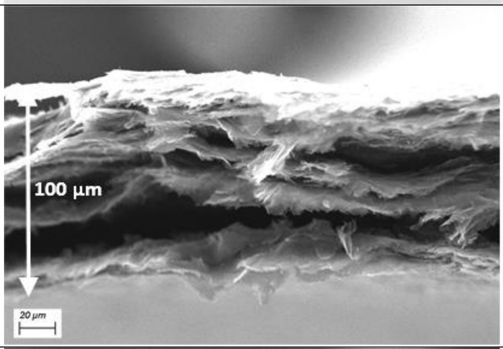	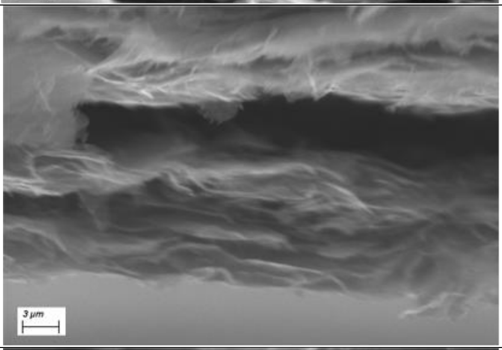
**PCL1-GNP1**	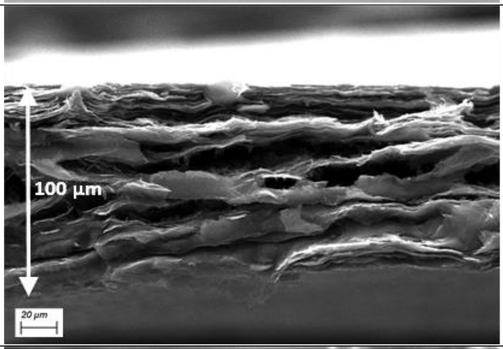	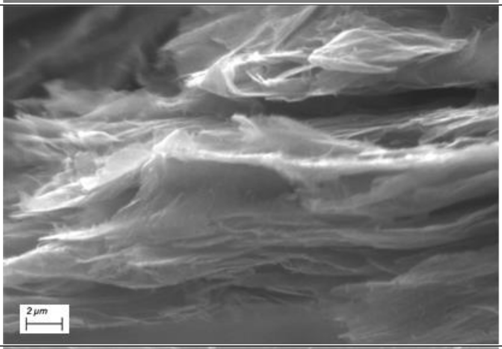
**PCL5-GNP1**	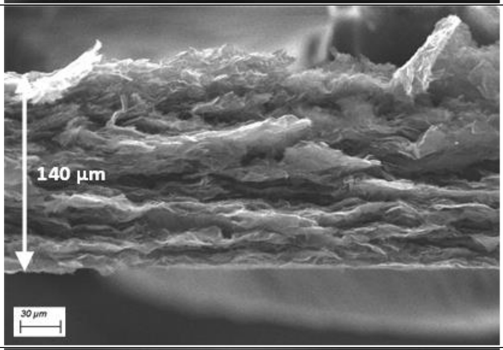	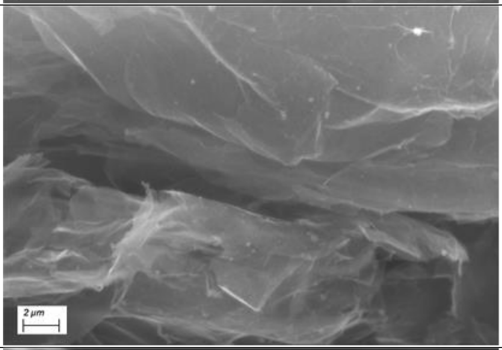
**PCL10-GNP1**	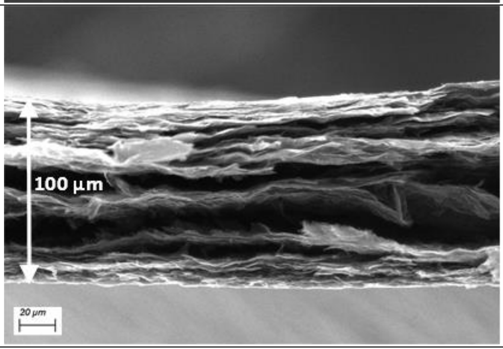	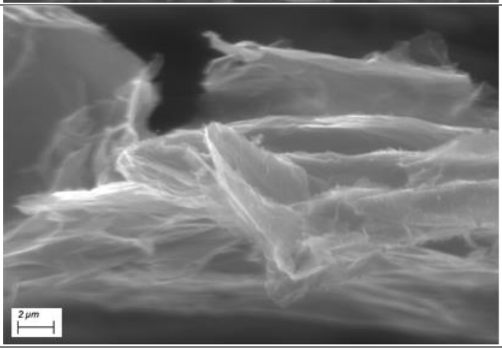

Figure S2. SEM micrograph for cross-section of different nanopapers, compressed at room temperature (cold-pressed), at different magnification and with approx. nanopaper thickness

	Low Magnification	High Magnification
**PCL1-GNP1-H**	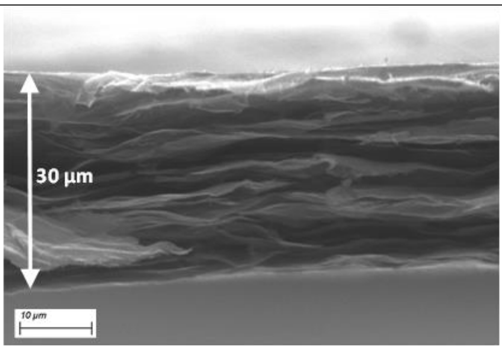	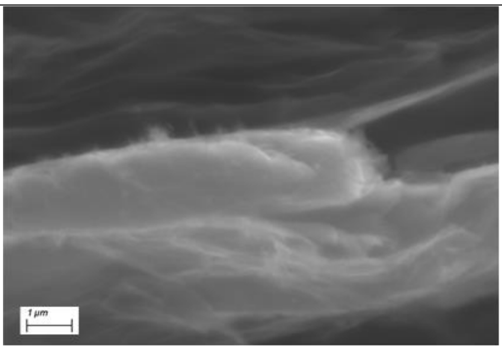
**PCL5-GNP1-H**	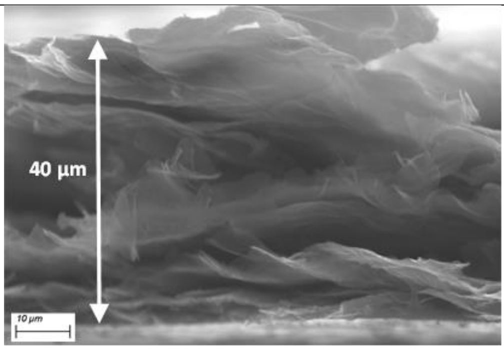	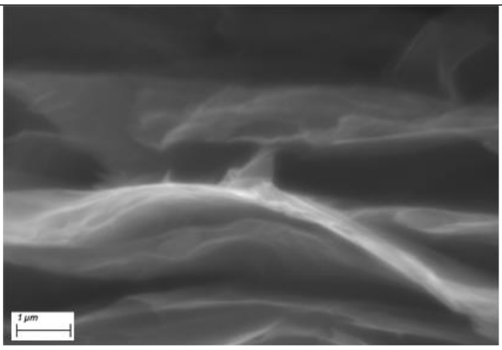
**PCL10-GNP1-H**	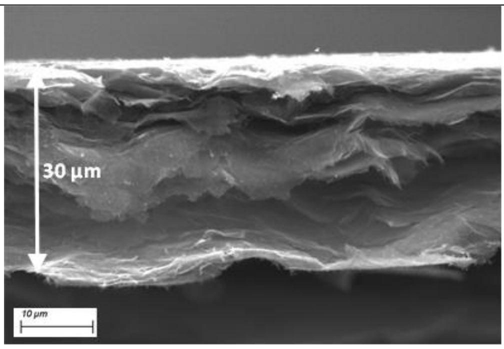	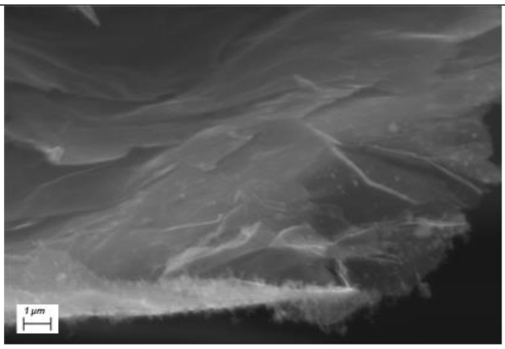

Figure S3. SEM micrograph for cross-section of different nanopapers, compressed at 80°C (hot-pressed), at different magnification and with approx. nanopaper thickness

Comparison between SEM micrographs for cold-pressed nanaopapers (Figure S2) and hot-pressed counterparts (Figure S3) clearly show differences in thickness, observable at low magnification (left images). Higher magnification (left images) further support higher compactness in hot-pressed films, as less voids are observed between GNP layers.

Section S3. PCL content

PCL content was evaluated by thermogravimetry, knowing GNP has a negligible mass loss up to 600 °C. Multiple tests were carried out on each formulation to average the possible inhomogeneities, yielding to average values and deviations reported Table 2. Representative TGA plots for the different nanopapers are reported in Figure S4 and Figure S5.

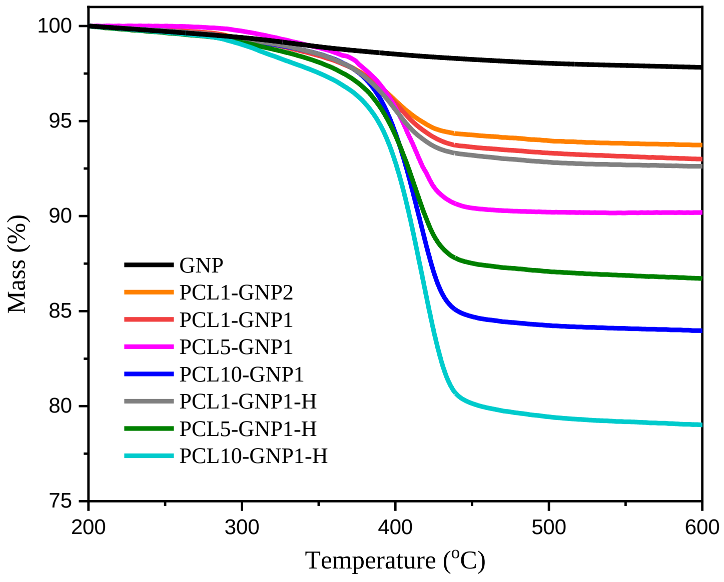

Figure S4. Mass plots vs. temperature from representative TGA test on different nanopapers formulation, as obtained.

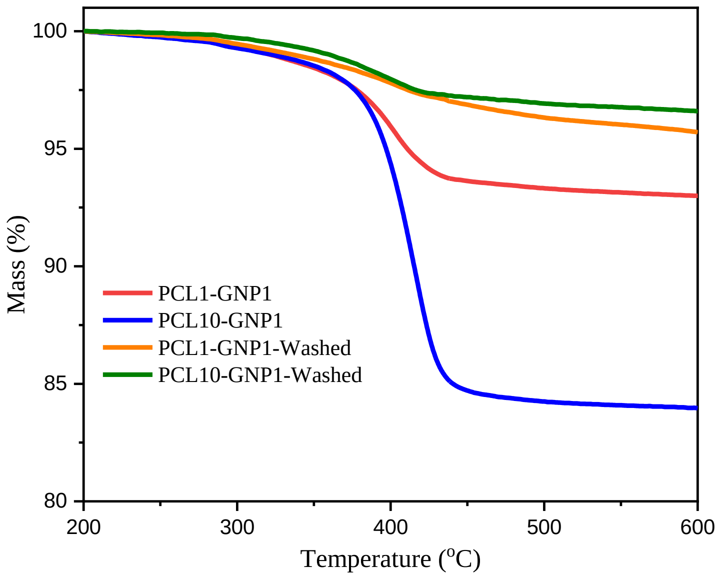

Figure S5. Mass plots vs. temperature from representative TGA test on different nanopapers formulation, as obtained vs. after extraction in toluene.

Section S4. Non-isothermal DSC experiments

DSC plots obtained on first heating for the different PCL-GNP nanopapers are reported in Figure S6.

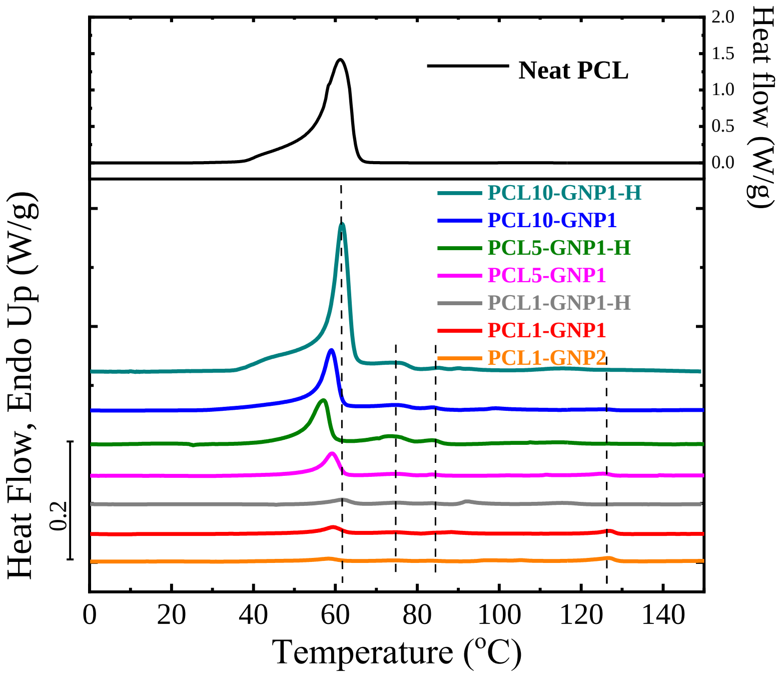

Figure S6. DSC curves for the first heating stage

Section S5. Unpressed vs. pressed samples

To investigate the origin of peak D, non-isothermal tests were performed in hot-pressed, cold-pressed and unpressed PCL5-GNP1, obtaining a smaller peak D in the hot-pressed samples compared to the cold-pressed or unpressed ones (Figure S7).

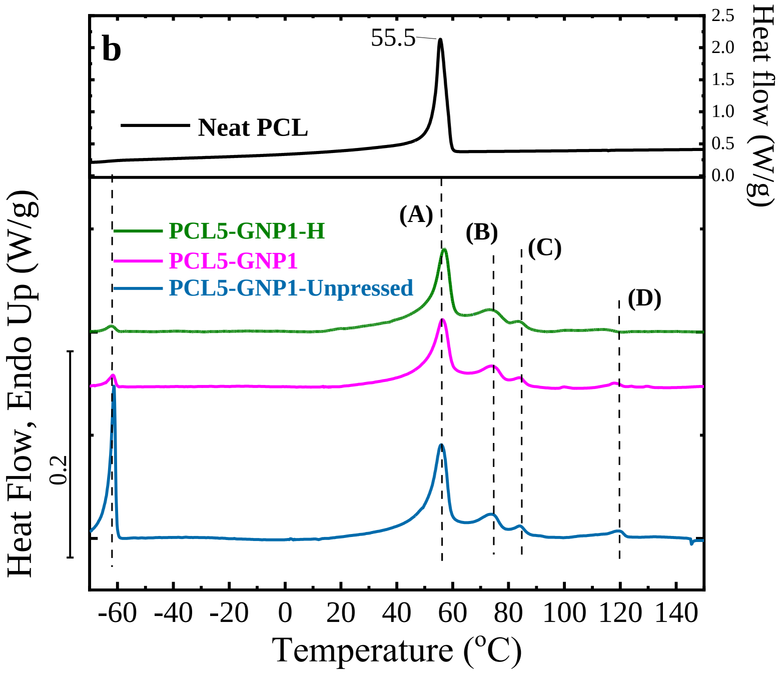

Figure S7. Comparison of the DSC second heating for cold-pressed, hot-pressed, and unpressed samples (PCL5-GNP1, PCL5-GNP1-H, and PCL5-GNP1-Unpressed).

Section S6. SSA results

This section presents additional comparisons of the final SSA heating scans on the different samples and comparisons of the final SSA heating and the corresponding second heating DSC scans obtained by non-isothermal tests. In Figure S8 we compare the final SSA heating scans of selected samples with different PCL contents.

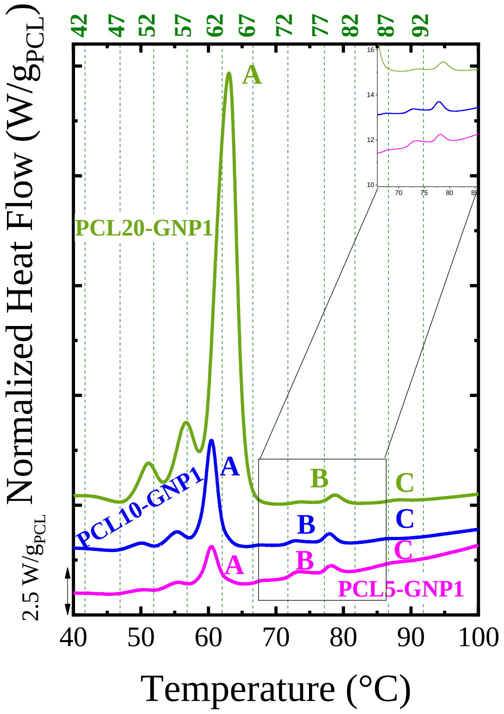

Figure S8. SSA Final Heating for PCL5-GNP1, PCL10-GNP1, and PCL20-GNP1. The green dashed lines indicate the fractionation performed at lower temperatures, with fractionation windows of 5 °C. The Ts employed are displayed at the top of the Figure. Note that the weight of PCL was used to normalize all the curves in each sample. The inset zooms-in the region of the "oriented PCL". The letters indicated the peak under fractionation according to Figure 2 in the main text.

Figure S8 shows that as the PCL content increases, peak A increases, as expected by the different PCL content. Interestingly, the opposite is observed for peak B (as is corroborated by the partial area analysis in Table S1). As a result, at lower PCL content, there is a higher interfacial area between the GNP surface and PCL chains, maximizing the influence of the GNP. Peak C is not susceptible to the SSA fractionation, a fact that may be explained if we consider that this peak is due to the melting of a pre-freeze PCL layer tightly bound to the GNP.

Figure S9 a to d shows the final SSA heating for all the samples. In all the cases, the indicated peaks A and B are the only ones that can be fractionated. Peaks C and D are not susceptible to be fractionated. Thus, peaks C and D maintain approximately the same position and area, during the final SSA heating, compared to the second heating of the non-isothermal experiments corroborating

that they are related to strongly absorbed PCL layers on the GNP surface. As a result, they are highly oriented and confined by the GNP, avoiding fractionation.

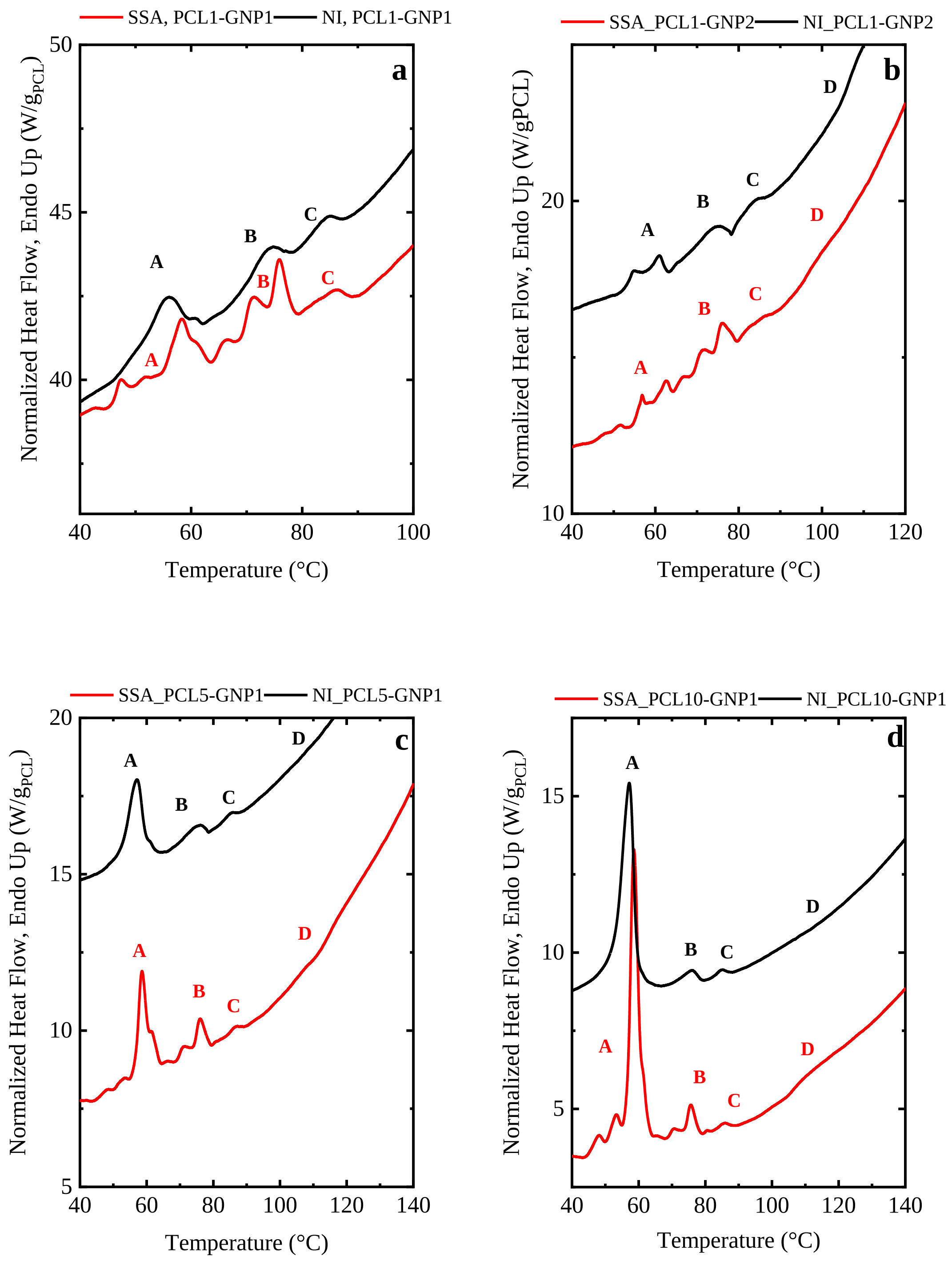

Figure S9. Comparison of the standard heating and the final SSA heating for (a) PCL1-GNP1, (b) PCL1-GNP2, (c) PCL5-GNP1, and (d) PCL10-GNP1.

Figure S10 compares the final SSA heating scan for the two selected samples to perform X-ray experiments (with the previous fractionation).

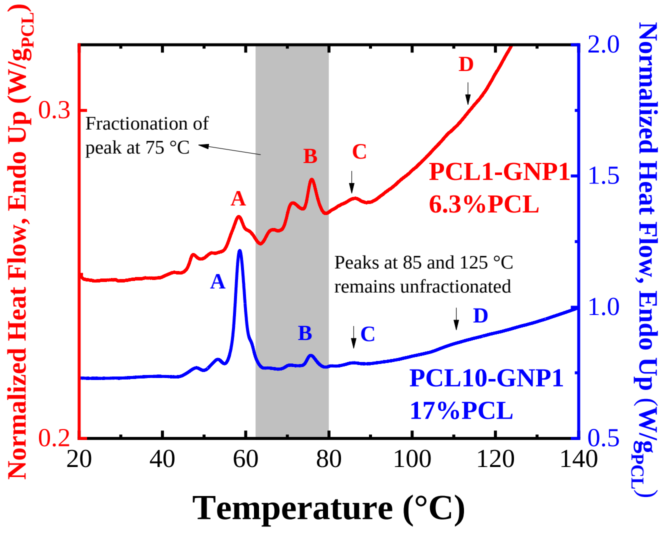

Figure S10. Comparison of the SSA final DSC heating scans for PCL10-GNP1 and PCL1-GNP1.

Figure S10 compares the SSA final DSC heating scans for two extreme samples. Only peak B (shadowed region in Figure S10) is susceptible to fractionation from the peaks at high temperatures. Interestingly, at high PCL content (PCL10-GNP1), the area of peak A is significantly higher than peak B, but at low PCL content (PCL1-GNP1) the inverse behavior is obtained.

Let us consider the unoriented (see peak A in Figure 5 and Figures S8 to S10) and oriented (see peak B and shadowed region in Figure 5a and Figure S10) PCL. The unoriented PCL is fractionated at T_s=42 to 57 °C (see Figure 5a and Figure S10) and the oriented PCL at T_s =62 to 82 °C. Considering these ranges, we calculated the partial areas of the endotherms. We found that as the ratio PCL/GNP decreases, the "oriented" PCL area increases (*e.g.*, 27.8% in the PCL10-GNP1 vs. 61.8% in the PCL1-GNP1), and the "unoriented" PCL area decreases (*e.g.*, 72.2% in the PCL10-GNP1 vs. 38.2% in the PCL1-GNP1). This indicates that the ratio PCL/GNP influences the PCL's absorption and orientation on the GNP. We quantify the partial area (from Figures S8 to S10) for all the cold-pressed samples, and the results are listed in Table S1. Further details related to the unoriented and oriented PCL fractions are shown in Section S9, and Figure S15.

Table S1. Partial areas obtained from SSA curves for the fractionated peaks A and B.

Sample	Partial Area, unoriented PCL or peak A (%)	Partial Area, oriented PCL or peak B (%)
PCL1-GNP1	38.2	61.8
PCL1-GNP2	24.0	76.0
PCL5-GNP1	63.2	36.8
PCL10-GNP1	72.2	27.8
PCL20-GNP1	92.2	7.8

Table S1 shows that as the PCL/GNP ratio increases, a lower proportion of oriented PCL is obtained.

We mentioned above that peak C remains unfractionated. To further prove this behavior, we have performed the SSA test with a fractionation window of 2.5 degrees to cover more points of peak C endotherm. The fractionation was conducted in the PCL1-GNP1 and PCL10-GNP1 samples, and the SSA final heating scans are shown in Figure S11.

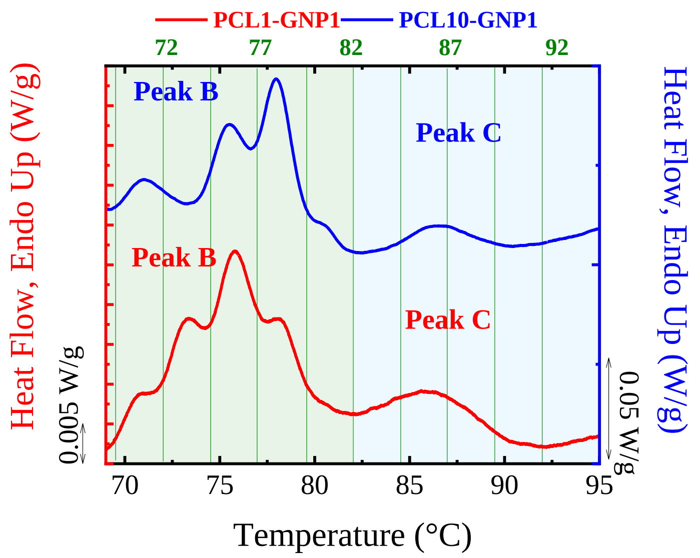

Figure S11. SSA final DSC heating scans for PCL10-GNP1 and PCL1-GNP1. The SSA protocol was conducted with a fractionation window of 2.5 degrees. The vertical lines indicate the different T_s values. As a guide, some T_s values are displayed at the top.

Figure S11 shows that peak B is fractionated according to the SSA steps every 2.5 degrees. In contrast, despite different SSA steps are performed in the melting range of peak C, it remains unfractionated. This confirms that peak C is highly oriented onto the GNP surface due to the pre-freezing phenomena.

Another interesting point is related to the fractionation profile of peak B. Figure S11 and Figure S12 show that the fractionation profile (peak B) changes as the PCL content in the sample decreases. More details are shown in Figure S12.

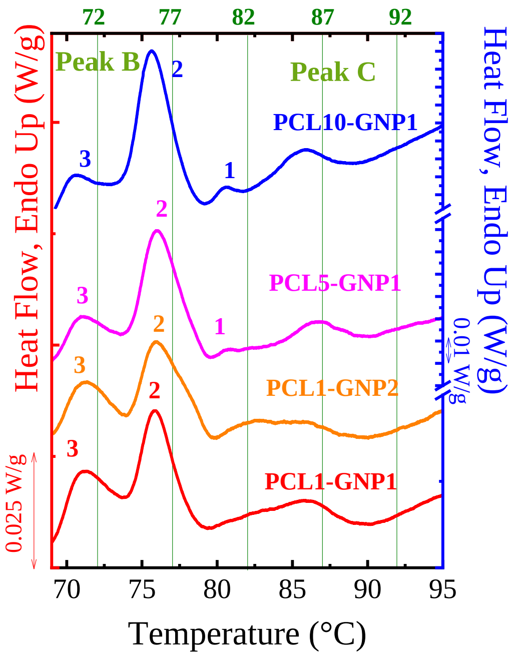

Figure S12. Comparison of the SSA profiles of peaks B and C for all the samples. The vertical lines indicate the different Ts (fractionation windows of 5 degrees), which are also labeled at the top.

Figure S12 shows a similar fractionation profile for peaks B and C of all the samples. As we already showed above, peak C remains unfractionated. For peak B, we have denoted its highest fraction as 1 and the lowest as 3. For PCL5-GNP1 and PCL10-GNP1, the fractionation profile (peak B) is similar, showing peaks 1 to 3, and a similar ratio between their areas. In contrast, for PCL1-GNP1 and PCL1-GNP2, peak 1 disappears, and the area of peak 2 is less significant (and the one of peak 3 is more significant) compared to higher PCL contents (*e.g.*, PCL10-GNP1). Thus, as we mentioned before, the samples exhibited different fractionation profiles. This can be attributed to the confinement experienced by the PCL crystal population (peak B). These results are similar to those previously found in PE-b-PS block copolymers as the PE content decreases in such strongly segregated copolymers.[1]

Section S7. WAXS analysis of peaks at high temperatures

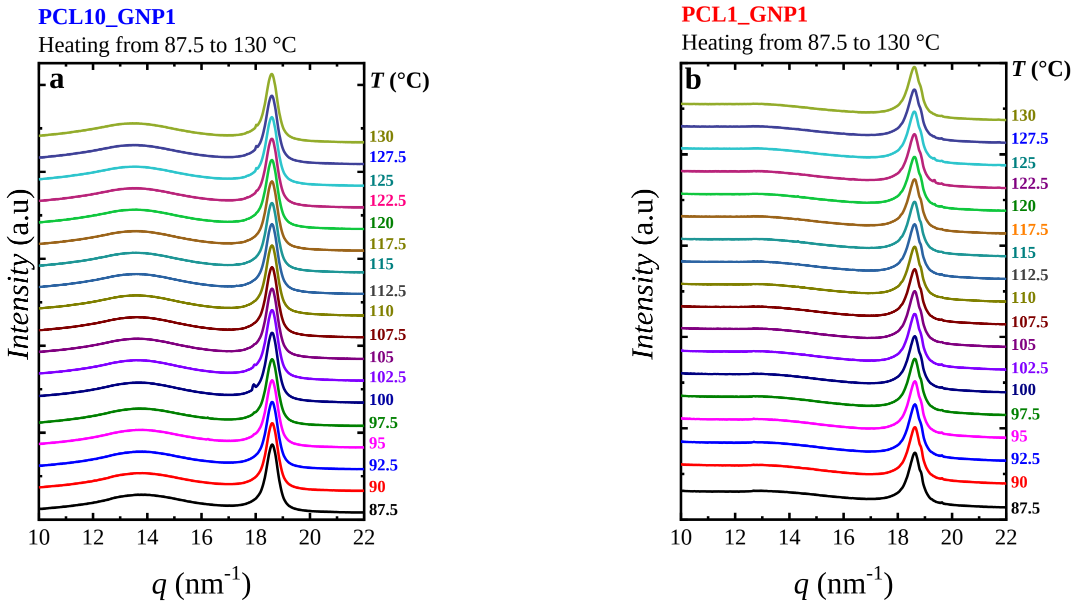

Figure S13. WAXS patterns taken during the SSA final heating from 87.5 to 130 °C for (a) PCL10-GNP1 and (b) PCL1-GNP1.

Figure S13 shows that in the range from 87.5 to 130 °C, the PCL is molten; hence, peaks C and D (see Figures 2 and 5 in the main text) cannot be detected by WAXS experiments due to their low diffraction volume.

Section S8. X-ray diffraction measurements (XRD)

XRD experiments were performed in the washed PCL10-GNP1 sample (washed with toluene by Soxhlet for one day, then dried overnight at 40 °C in a vacuum), in which only peak D was detected by DSC. The sample was analyzed in a Philips PW 1830 powder diffractometer (Ni-filtered Cu Ka radiation, k = 0.1542 nm). XRD equipment, in the range from 10 to 60°. Figure S14 shows the XRD curve, which revealed the presence of the characteristic strong reflections of the GNP at 2θ angles of ~26° (d_{002}=0.342 nm) and 55° (d_{004}=0.167 nm), indexed to the *(002)* and *(004)* planes of the graphite. Medium-intense reflections at 2θ angles of ~ 40° (d_{110}=0.225 nm), 44° (d_{101}=0.206 nm), 48° (d_{102}=0.189 nm), and 58° (d_{103}=0.159 nm) are in good agreement to the *(100)*, *(101)*, *(102)* and (103) planes of the graphite.[2, 3]The small reflections at 2θ= 21.7 (d_{110}=0.409 nm) and 24.1° (d_{200}=0.369 nm) are related to the *(110)* and *(200)* principal planes of the PCL.[4] Interestingly, the intensity for the *(200)* have a comparable intensity with the *(110)*, suggesting a different PCL organization. The intensity of the remaining signals (see an asterisk in Figure S14) is higher than the main reflections of the PCL; thus, these signals correspond to impurities, likely within the graphite used as raw material for the production of GNP.

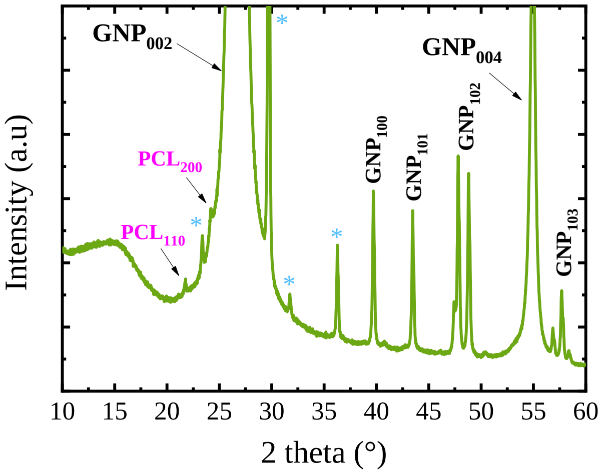

Figure S14. XRD for washed PCL10-GNP1. The position of the different peaks corresponding to PCL and GNP is indicated. The signal corresponding to impurities is marked with an asterisk.

Section S9. Orientation in PCL1-GNP1 and PCL10-GNP1 samples

According to the previous results, we proposed that a fraction of the PCL is oriented along the GNP, whereas other fractions remains unoriented. To prove this hypothesis, we performed WAXS experiments using a transmission configuration to determine the orientation in the PCL10-GNP1 and PCL1-GNP1 nanopapers. Figure S15a shows the WAXS patterns for these samples, and Figure S15b illustrates the azimuthal profile for the PCL10-GNP1.

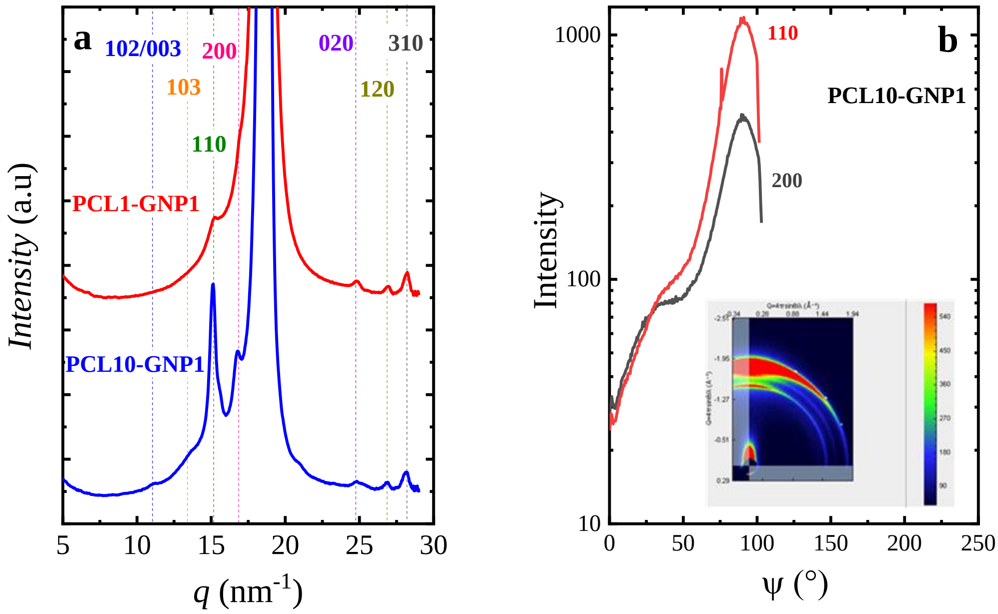

Figure S15. (a) WAXS patterns for PCL1-GNP1 and PCL10-GNP1, and (b) Intensity distribution of selected reflections vs. azimuthal angle for PCL10-GNP1 obtained from the reflections of the planes *(110)* and *(200)*, detected in the inserted image.

In Figure S15a, besides the planes detected in Figure 6 (manuscript), we have detected the reflections corresponding to the planes *(020)*, *(120),* and *(310)*. As in Figure 6, the PCL10-GNP1 shows stronger intensity than the PCL1-GNP1 due to its higher crystallinity. In Figure S15b, we show the azimuthal profile of the PCL10-GNP1, which evidences the orientation on the sample, corroborating the existence of unoriented and oriented PCL fractions in the nanopapers.

Section S10. References

1. Lorenzo, A. T.; Arnal, M. L.; Müller, A. J.; Boschetti de Fierro, A.; Abetz, V., Confinement Effects on the Crystallization and SSA Thermal Fractionation of the PE Block within PE-b-PS Diblock Copolymers. *Eur. Polym. J.* **2006,** *42* (3), 516-533.
2. Li, Z. Q.; Lu, C. J.; Xia, Z. P.; Zhou, Y.; Luo, Z., X-ray Diffraction Patterns of Graphite and Turbostratic Carbon. *Carbon* **2007,** *45* (8), 1686-1695.
3. Wang, Y.; Panzik, J. E.; Kiefer, B.; Lee, K. K. M., Crystal Structure of Graphite under Room-temperature Compression and Decompression. *Sci. Reports* **2012,** *2* (1), 520.
4. Bittiger, H.; Marchessault, R. H.; Niegisch, W. D., Crystal Structure of Poly-ε-caprolactone. *Acta Crystallograph. B* **1970,** *26* (12), 1923-1927.
